# Annexin-V binds subpopulation of immune cells altering its interpretation as an *in vivo* biomarker for apoptosis in the retina

**DOI:** 10.7150/ijbs.102551

**Published:** 2024-11-11

**Authors:** Kiyoharu Joshua Miyagishima, Francisco Manuel Nadal-Nicolás, Wenxin Ma, Wei Li

**Affiliations:** Retinal Neurophysiology Section, National Eye Institute, NIH, Bethesda, MD, USA.

**Keywords:** annexin, biomarker, immune cells, DARC, *in vivo* imaging, optic nerve injury

## Abstract

In cells undergoing apoptosis phosphatidylserine, a major component of the plasma membrane, translocates to the outer leaflet where it provides eat-me signals for phagocytic recognition and is bound by annexin-V, an apoptotic marker. The need to track retinal ganglion cell death (RGC) in response to glaucomatous damage or optic neuropathy has led to the development of DARC (detection of apoptosing retinal cells) imaging, providing non-invasive, *in vivo* assessment of RGC death. Although the eye is an immune privileged site, resident and infiltrating immune cells are known to respond quickly to trauma or infection. Some immune cells have binding sites for annexin homologs; thus, their presence may confound estimates of apoptosis measured by annexin-V labeling. The purpose of this study was to re-examine the accuracy of annexin-V apoptotic labeling in the posterior eye and to temporally characterize contributions of non-apoptotic labeling in response to optic nerve (ON) injury. Here, we found annexin-V labeling consists of two phases. Initially, there is a rapid phase matching the time course of apoptotic cell death indicated by cleaved caspase-3 immunostaining observed *ex vivo*. This is followed by a sustained plateau phase that persists long after the peak of degeneration. We demonstrate that annexin-V binds to a specific subpopulation of myeloid cells in the retina, which were identified using simultaneous confocal scanning laser ophthalmoscopy. Optical coherence tomography and confocal imaging reveal these cells occupy the posterior hyaloid space above the retinal nerve fiber layer and at various retinal depths. Our results highlight the cellular morphological heterogeneity of non-apoptotic annexin-V labeling of retinal microglia. Accordingly, pharmacological depletion of microglia abolishes annexin-V labeling of elongated microglia *in vivo* revealing fainter labeling of round RGCs. Thus, consideration should be given to the time course of the immune response when interpreting fluorescently labeled annexin-V to visualize retinal cell apoptosis for clinical diagnosis.

## Introduction

Annexin-V is a commonly used apoptotic marker that selectively binds to exposed phosphatidyl serine when cells undergo apoptosis 1-3. In glaucoma or following optic nerve (ON) injury, RGCs of the retina undergo apoptosis leading to blindness. Thus, DARC (Detection of Apoptosing Retinal Cells) imaging was developed to use annexin-V to visualize and track apoptosis of retinal cells *in vivo,* taking advantage of the eye's transparency using conventional confocal scanning laser ophthalmoscopy (cSLO) 4. The technique has been applied to various eye diseases such as glaucoma 5-11 and advanced over the years with the addition of AI-assisted counting and is now being used in clinical trials 11-13.

However, monocytes and neutrophils possess binding sites for annexin homologs, like annexin-I 14. Although annexin-V and annexin-I differ in thermal stability 15, they share Ca^+^ dependent phospholipid binding and are remarkably similar in structure 16-18. These conflicting results reflect heterogeneity in the labeling of cells with annexin and called for a closer investigation of which cell types possess annexin-V labeling in the retina. To address this, we assessed annexin-V co-labeling in CX3CR1^GFP/+^ mice, a popular microglia reporter line 19, in an ONC injury model. Our findings reveal that even in naïve retinas, annexin-V labels subsets of both CX3CR1^+^ and CX3CR1^-^ myeloid cells in both the mid and far periphery and at the ON head. In addition to apoptotic retinal cells, vitreous cells contribute to annexin-V^+^ labeling following injury. These myeloid cells, likely hyalocytes, matched the pattern of annexin-V labeling obtained using cSLO and were also visible in the vitreous above the plane of the retina in OCT images acquired simultaneously. Some microglia became annexin-V positive after phagocytosing dying RGCs. Annexin-V^+^ cells can be subdivided based on morphology (ameboid/round vs elongated) revealing disparate time course of cell death and myeloid cell expansion either by proliferation or infiltration. Pharmacological depletion of microglia in naïve and in ONC retinas reveal that the faint labeling of apoptosing RGCs is normally masked by the bright annexin-V labeling of these microglia.

## Results

Using a cSLO we imaged naïve WT mice after intravitreal injection of annexin-V to determine appropriate concentrations for improved detection. Intravitreal delivery was selected to provide annexin-V directly to the retina avoiding potentially undesirable systemic effects. Our results indicate no difference in the number of cells labeled between the low dose (0.05µg/µl, n=4 retinas, **Fig. S1 A-C**) versus the high dose (0.2µg/µl, n=4 retinas, **Fig. S1 D-F**) annexin-V (**Fig. S1 G**). However, in some instances we observed slight leakage upon removing the syringe. To image both bright and weakly labeled cells and to ensure uniform distribution of annexin-V we opted to perform the following studies using the higher concentration of 0.2µg/µl delivered in a volume of 1.25µl. The amount of annexin-V delivered (0.25µg) into the vitreous chamber is comparable to previously published reports for the use of DARC imaging in mice (0.5µg) 20 allowing for direct comparison.

### Time course of annexin-V labeling *in vivo* does not fully match rate of retinal cell apoptosis

**Figure 1:**
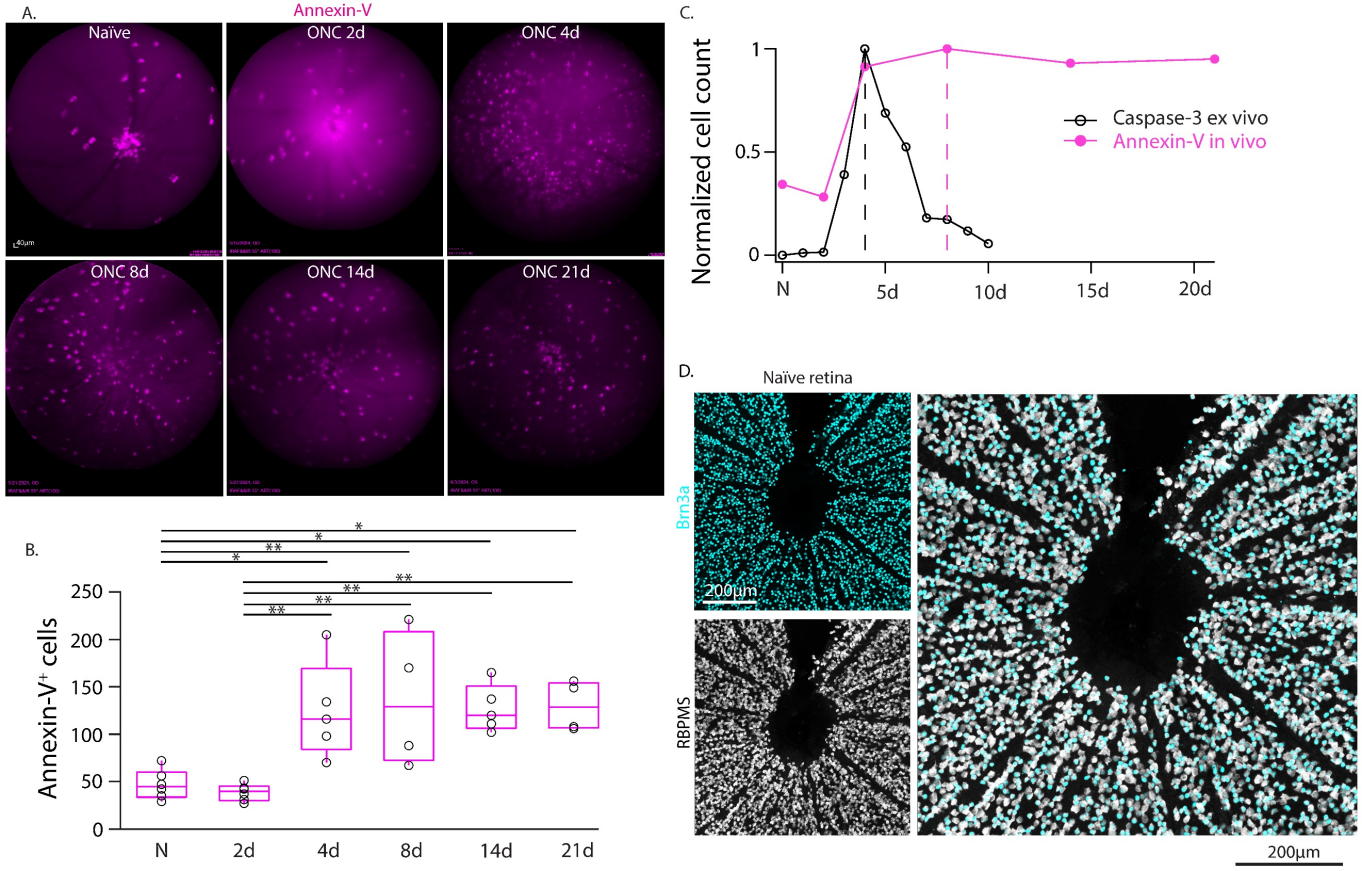
Longitudinal imaging of annexin-V labeling of the retina in WT mice *in vivo* in intact retinas and following optic nerve crush injury. **A.** cSLO fundus images (n=4 mice) taken at regular intervals (0d, 2d, 4d, 8d, 14d, 21d) up to 3-weeks post injury. This longitudinal study was performed on the same eyes from a group of animals that were analyzed at all time points (and collected for anatomy at 21d). Scale bar: 40µm. **B.** Boxplot graphs of the labeling quantification of annexin-V signals from the cSLO fundus images. Black line in the middle of the boxplots indicates the median. One-way ANOVA with Tukey's multiple comparisons post-hoc test was performed. * indicates p<0.05, **<0.01. **C.** Comparison of the time course of annexin-V and caspase-3 (adapted from Sánchez-Migallón et al., 2016) 21 labeling after optic nerve crush. Note: The caspase-3 labeling and quantification is taken from whole retinas after dissection whereas the annexin-V labeling is imaged *in-vivo*. Cleaved caspase-3 labeling (a cell death marker) peaks at 4 days (dashed line) and declines thereafter whereas the annexin-V labeling peaks at 8 days (dashed magenta line) and only slightly decreases even up to 21 days. The temporal activation of caspase 3 21 (https://iovs.arvojournals.org/article.aspx?articleid=2482663) is plotted for comparison unmodified and is available under public license under the terms of the Creative Commons Attribution 4.0 International License (https://creativecommons.org/licenses/by/4.0/). **D.** Confocal image of retinal ganglion cells labeled with Brn3a (cyan) and RBPMS (white) surrounding the ONH. Scale bars: 200µm.

We used this technique to perform real-time *in-vivo* imaging of annexin-V in WT mice and tracked the labeling of annexin-V^+^ cells over time following optic nerve crush injury (**Fig. 1A-B, Table S1**). We noted that the time course of annexin-V^+^ cells did not match the expected time course of cleaved caspase-3 mediated neuronal loss 21 following optic nerve crush (**Fig. 1C**) which peaks at 4 days and declines thereafter. The number of annexin-V^+^ cells similarly increase at 4 days but peak at day 8 and remain elevated even up to 21d. We were also surprised to find annexin-V labeled cells in the naïve retina concentrated at the optic nerve head, a region devoid of retinal ganglion cells (**Fig. 1D**). Thus, we questioned whether *in vivo* use of annexin-V is an exclusive biomarker for apoptosis.

### Annexin-V^+^ cells visualized *in vivo* co-localize with a subset of CX3CR1^+^ retinal microglia

**Figure 2:**
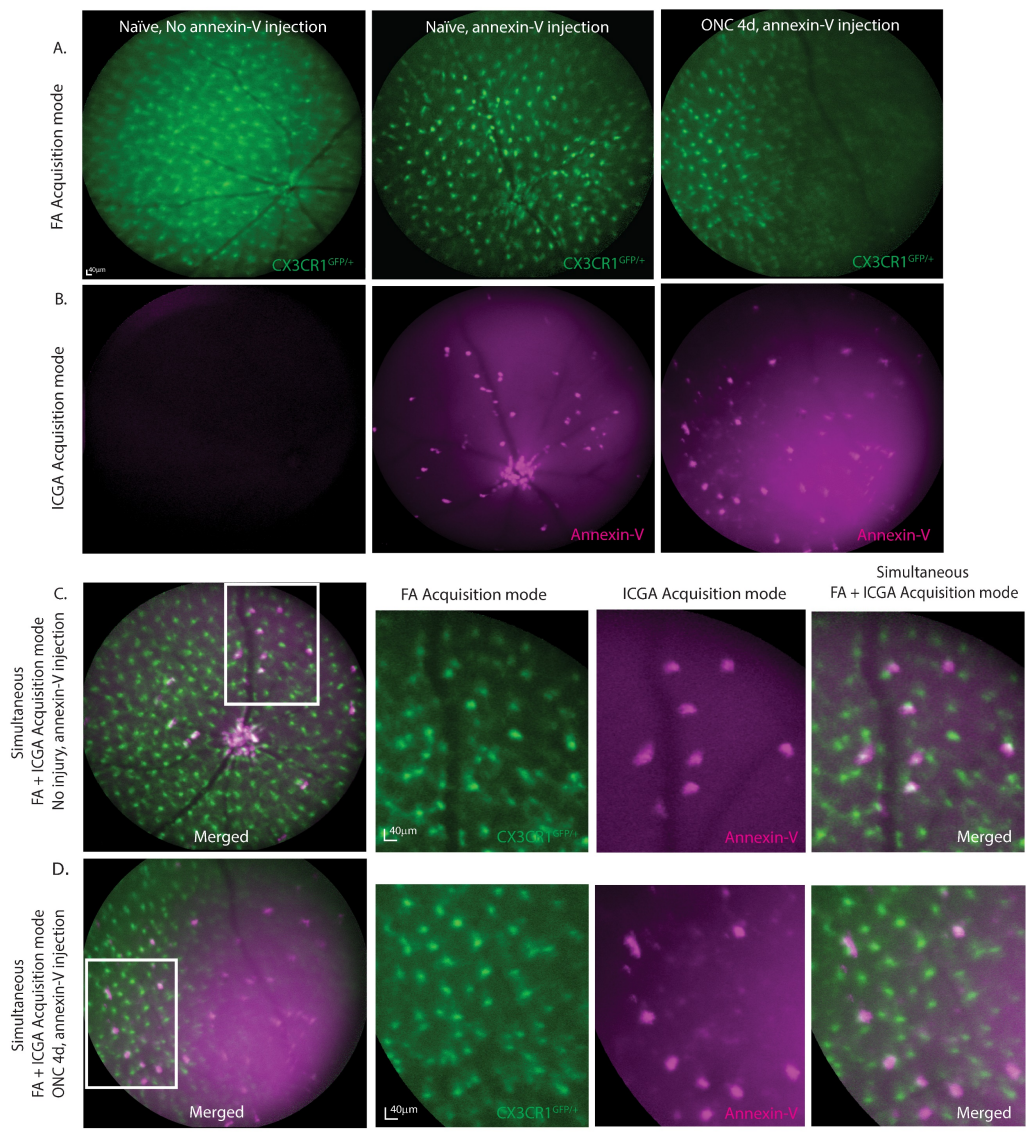
Fundus fluorescence imaging at 488nm and 790nm shows co-labeling of annexin-V with subpopulation of cells positive for CX3CR1. **A.** cSLO fundus image (FA acquisition mode) of CX3CR1^GFP/+^ mouse retinas with or without annexin-V (CF@800) injection. **B.** Near-infrared fundus fluorescence images of annexin-V labeling with or without ONC injury. Some annexin-V^+^ cells are located at the ONH and vasculature. **C.** Simultaneous FA+ICGA acquisition mode of CX3CR1^GFP/+^ control mouse and **D.** 4d after ONC, zoom images of annexin-V co-labeling with CX3CR1 shown on the right, co-labeled cells resemble immune cell morphology. Scale bars: 40µm.

To determine whether annexin-V binds to other cell types such as retinal microglia, we simultaneously acquired *in vivo* images of annexin-V positive cells in heterozygous CX3CR1 knockin mice (CX3CR1^GFP/+^) that expressed enhanced green fluorescent protein in the CX3CR1 loci of microglial cells. Using a cSLO, fundus images were acquired simultaneously using both the 790nm and 488nm laser to excite annexin-V and CX3CR1^GFP/+^ respectively. Microglial cells are predominantly found in the inner plexiform layer (IPL), outer plexiform layer (OPL), and the retinal nerve fiber layer (RNFL) 22 where they adopt different morphology depending on their activity states. Microglia in their surveillance state appear ramified and form a mosaic network across the retina. Upon activation in response to injury or trauma such as ONC, microglia are activated and changed to round, ameboid-shape to move to the site of injury where they secrete inflammatory cytokines and phagocytose dead or dying cells/debris 23. The CX3CR1^GFP/+^ expression consistently identifies and locates retinal microglial cells (**Fig. 2A**). Without annexin-V, no fluorescence is observed at 790nm (**Fig. 2B, left**). Without annexin-V, no fluorescence is observed at 790nm (**Fig. 2B, left**). Intravitreal annexin-V injection labels cells at the ONH and sparsely throughout the retina even without injury (**Fig. 2B, middle**). At ONC4d, annexin-V labeling becomes more distinct showing both bright and faint spots (**Fig. 2B, right**). Bright spots co-localize with a subset of CX3CR1-GFP positive cells (**Fig. 2C**), while the rounder faint labeling likely indicates retinal apoptotic cell death (**Fig. 2D, Fig. S2A-B**). While CX3CR1 is widely expressed in retinal microglia, it is also expressed in some monocytes infiltrating from the blood 24.

### OCT confirms presence of cellular infiltrates in the vitreous that are annexin-V^+^

**Figure 3:**
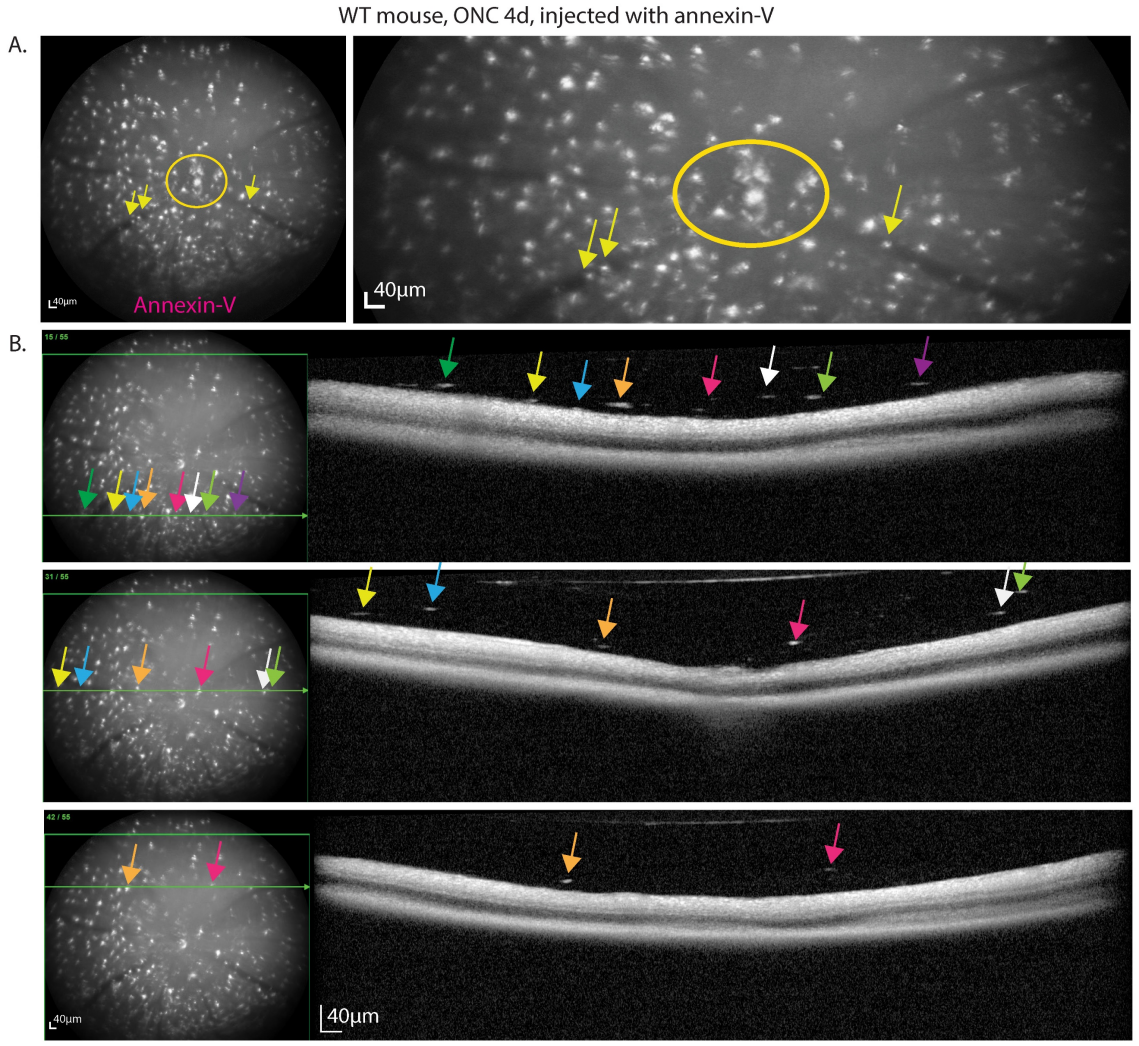
DARC imaging of mouse retina at 4d post ONC reveals labeling of immune-like cells in the vitreous. **A.** Cells labeled with annexin-V can be seen in the ONH (circle) and in the vasculature (arrows), regions devoid of RGC somas. **B.** Fundus and OCT b-scan (green line) that maps the bright annexin labeling from the fundus to the hyperfluorescent spots observed in the vitreous above the retinal nerve fiber layer that are larger/denser than speckle noise. Scale bars: 40µm.

Only a small subset of CX3CR1-GFP positive cells were annexin-V^+^ suggesting that these cells are distinct from majority of microglia (annexin-V^‑^) that tile the retina. cSLO fundus images of the retina ONC4d injected with annexin-V reveal hyperfluorescent bright spots, especially clustered at the optic nerve head (ONH) where RGC axons converge to exit the eye to convey visual information to the brain. The ONH (circle) and retinal vasculature (arrow) lack RGCs, suggesting that annexin-V binding is not exclusively indicative of apoptosing RGCs (**Fig. 3A, Fig. S2C**).

The small white hyperfluorescent spots from annexin-V in the cSLO fundus images co-localize with dense particles in the vitreous in OCT images (**Fig. 3B inferior, central, superior**). In addition to dying RGCs, this suggests that many annexin-V labeled cells are likely CX3CR1-GFP^+^ immune cells or migrating vitreal cells 25 in response to injury, possibly indicating inflammation. Notably, liquid based cytology identified vitreous cell composition to include CD68^+^ macrophages, retinal pigment epithelium (RPE), and DEC-205^+^ dendritic cells in various retinal pathologies 26.

**Figure 4:**
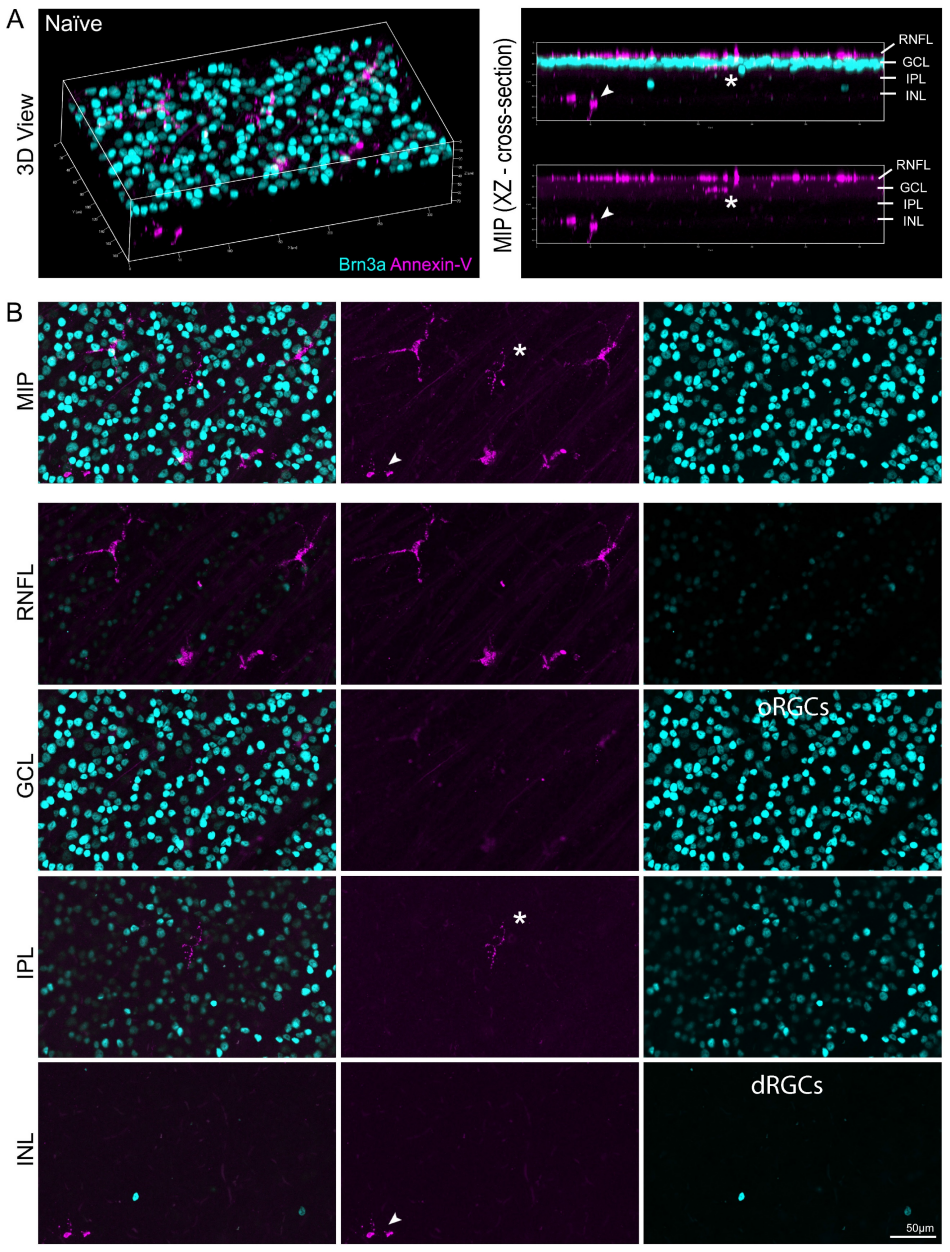
*Ex vivo* confocal image of naïve retina showing location of annexin-V labeled cells within the retinal layers. **A.** (Left panel) Naïve mice were intravitreally injected with annexin-V 1h prior to collecting the eyes for fixation and immunohistochemistry. Dissected retinas were stained for Brn3a (retinal ganglion cell marker, cyan). (Right panel) Cross sectional image showing annexin-V^+^ cells are primarily located on or in the retinal nerve fiber layer. **B.** Image Z-stack was separated to isolate different retinal layers. Asterisk and arrow indicate annexin-V^+^ cells that are found in the inner retina. Orthotopic retinal ganglion cells: oRGCs. Displaced retinal ganglion cells: dRGCs. Scale bar: 50µm.

We confirmed our OCT findings by confocal microscopy to analyze annexin-V^+^ cell distribution in the retinal layers**. Fig. 4A (left)** shows a 3D view of a naïve retina labeled with annexin-V (magenta) and brain-specific homeobox/POU domain protein 3 (Brn3a, cyan). The cross section (**Fig. 4A, right**) reveals that most of the annexin-V^+^ cells are in the RNFL, but some are also in the inner retina, indicating that the dye penetrated deeper retinal layers. The maximum intensity projection provides an en face view of annexin-V labeled cells in this area (**Fig. 4B, top**). The asterisk and arrow highlight annexin-V^+^ cells deeper in the retina. Annexin-V^+^ cells are prominently visible in the RNFL, IPL, and in the INL alongside displaced retinal ganglion cells.

### Confocal images identify annexin-V^+^ subpopulation of myeloid cells with varied morphology

**Figure 5:**
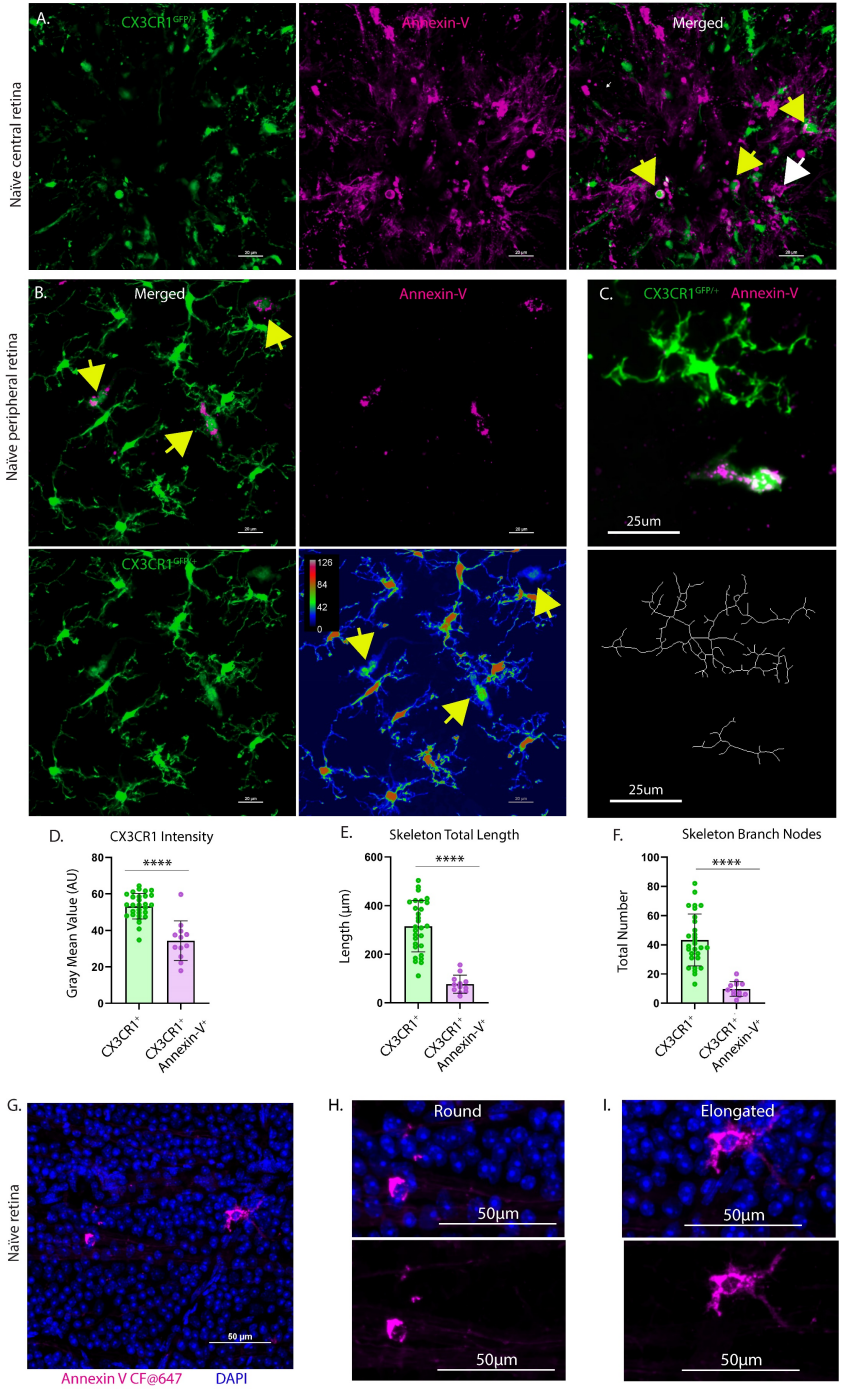
Retina explants from CX3CR1^GFP/+^ mice intravitreally injected with annexin-V. **A.** CX3CR1-GFP positive (yellow arrows) and CX3CR1-GFP negative (white arrow) myeloid cells appear to reside or enter the retina through the ONH. **B. (**upper left**)** In naïve retinas, annexin-V colocalizes (yellow arrows) with CX3CR1^+^ cells. **B.** (lower right) Image replotted using a pseudocolor look-up table assigning colors to each pixel based on original brightness value. Annexin-V^+^ microglia have weaker CX3CR1 expression (yellow arrows) compared to annexin-V^-^ microglia. For **A-B**, Scale bars: 20µm. **C.** (Top) Cropped confocal image of annexin-V^-^ and annexin-V^+^ microglia. (Bottom) Skeletons of their microglial morphology. Scale bars: 25µm. **D.** Quantification of gray mean value of CX3CR1 intensity, **E.** skeletal total length in microns, and **F.** number of skeletal branch nodes for different populations of microglia (n=8 images, n=29 CX3CR1^+^, n=12 CX3CR1^+^ Annexin-V^+^). Error bars indicate SD. ****p<0.0001, statistically significant change (Student's t-test). **G.** Confocal image of naïve retina counterstained with DAPI to verify that the annexin-V signals correspond to cells that have intact nuclei (eliminating the possibility of misinterpreting it as unconjugated/unbound annexin-V dye or cells undergoing apoptosis). **H.** Zoomed image showing annexin-V^+^ cell with ameboid morphology. **I.** Zoomed image showing annexin-V^+^ cell with elongated morphology. For **G-I**, Scale bars: 50µm.

To further confirm the heterogeneity of annexin-V binding in the retina we injected annexin-V intravitreally and analyzed retinal whole mounts by confocal microscopy. As in the cSLO images, annexin-V labeled a subset of CX3CR1-GFP positive (**Fig. 5A-B**) and CXCR1-GFP negative (**Fig. S2D**) myeloid cells primarily in the nerve fiber layer (**Fig. 5A**). Annexin-V^+^ cells generally exhibited fainter CX3CR1 expression (**Fig. 5B, 5D**) suggesting activation, compared to neighboring CX3CR1^+^ microglia, which had more branching and arborization (**Fig. 5C, 5E-F, S2C**). Cells near the ONH may be infiltrating monocytes that can differentiate into macrophages and dendritic cells 27. Thus, we conclude that annexin-V labels subsets of myeloid cells. CX3CR1 expression appears to delineate different lineages or different stages of myeloid development indicating distinct roles in maintaining the retina environment.

### Round annexin-V^+^ cells match rapid time course of apoptosis whereas elongated annexin-V^+^ cells contribute to sustained plateau

**Figure 6:**
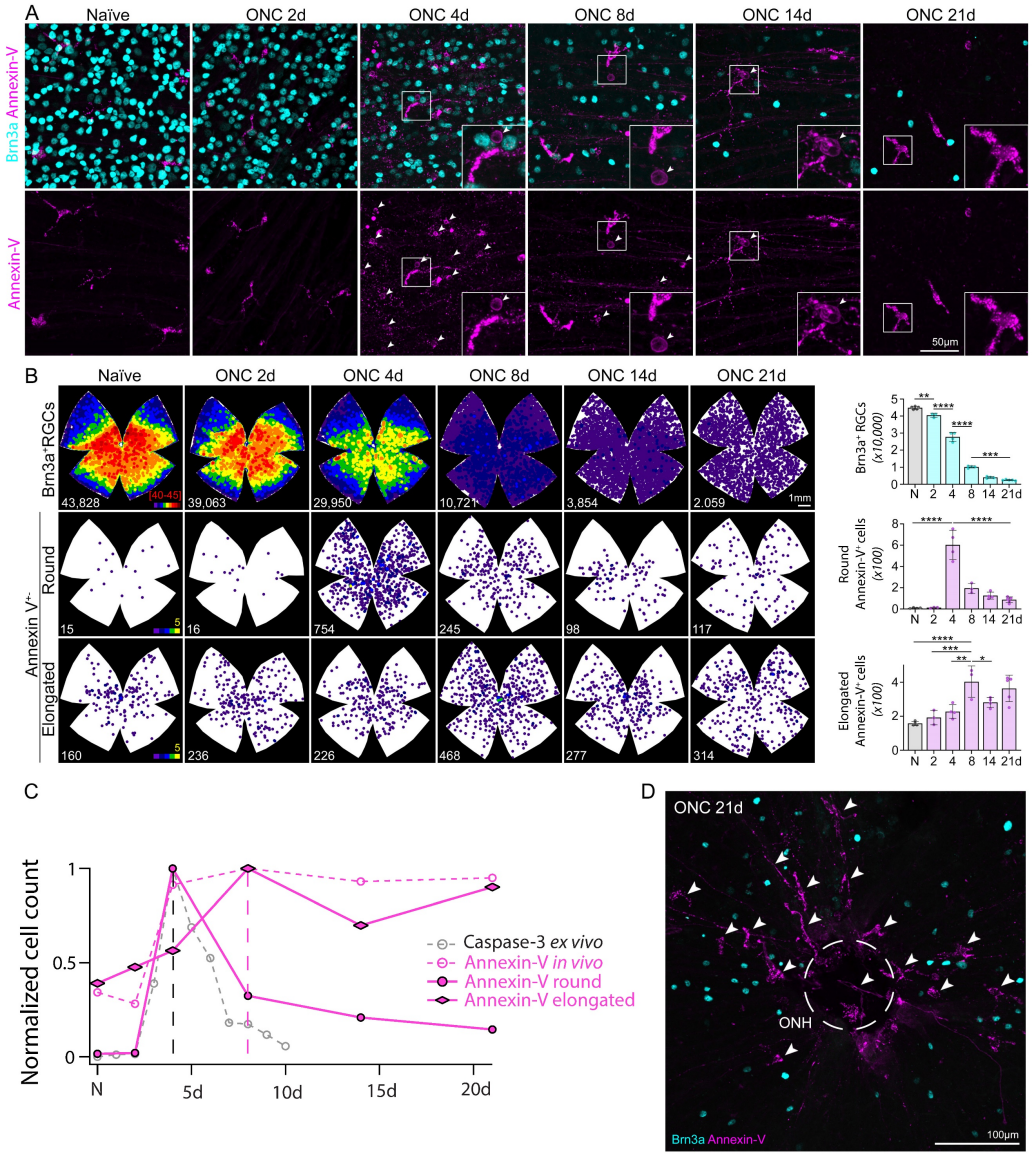
Time course of RGC (Brn3a, cyan) degeneration and annexin-V^+^ cells subdivided by morphology. **A.** Images show Brn3a^+^ RGCs and annexin-V in naïve retinas and following ONC 2d, 4d, 8d, 14d, and 21d. This group of mice were evaluated *in vivo* longitudinally at different time points corresponding to the images shown in Figure 1. Arrows indicate annexin-V^+^ retinal ganglion cells because of their round morphology, and faint staining which is mostly localized to the cell membrane. In some cases, the cells are recognizable as RGCs because the annexin-V also binds to their dendrites toward the INL (e.g. insert of ONC 14d). **B.** (Left) Isodensity maps showing the distribution of RGCs and annexin-V^+^ cells classified by morphology (elongated vs. round). **B.** (Right) Quantification of surviving RGCs and annexin-V^+^ cells with either round or elongated morphology. **C.** Plot normalized number of annexin-V^+^ cells with round or elongated morphology following optic nerve crush overlayed onto **Fig. 1C** to compare the time course with annexin-V labeling *in vivo* and caspase-3 labeling *ex vivo*. **D.** Image of central region of whole mount retina ONCd21 labeled with Brn3a (cyan) and annexin-V (magenta). Encircled region (dashed lines) indicates optic nerve head (ONH).

To further characterize the heterogeneity of these annexin-V positive cells we subdivided them based on morphology (ameboid/round vs elongated, **Fig. 5 G-I**) enabling us to track and quantify changes in cell number in relation to the rapid RGC degeneration associated with ONC. WT mice were subjected to ONC and the retinas were collected at varying time points (0d, 2d, 4d, 8d, 14d, 21d) and stained for Brn3a to label RGCs. The number of Brn3a^+^ cells was quantified semi-automatically using ImageJ 28. As expected, the temporal course of surviving Brn3a^+^ RGCs (**Fig. 6A top, Fig. 6B top, Table S2**) matched previously reported studies using RBPMS and other RGC markers 29. Cells classified by their round annexin-V^+^ morphology peaked at 4d (**Fig. 6A bottom**, **Fig. 6B middle, Table S2**) and had a similar time course to cleaved-caspase 3 labeling 21 (**Fig. 6C**) suggesting that annexin-V^+^ cells rounder in appearance were likely apoptotic RGCs.

We also classified a population of myeloid cells that were annexin-V^+^ but had elongated morphology (**Fig. 6B, bottom**) and do not seem to respond to the earlier stages of RGC degeneration. These cells peaked in number at 8d (**Fig. 6C**) which is somewhat striking as most of the cell death has already occurred by this time (23% surviving Brn3a+ RGCs, **Fig. 6B top**), implying a different role than those involved in inflammation 23. At day 21, elongated annexin-V^+^ cells (arrows) are visible around the ONH (**Fig. 6D**) and appear to fill in the open free space left by the death of RGCs. Assuming they are of myeloid cell lineage they would form a cellular network that shapes the adaptive immune response after the initial wave of RGC death, perhaps even attempting to restore retinal homeostasis or prevent secondary neuronal degeneration.

### Pharmacological depletion of microglia eliminates bright annexin-V labeling in cSLO images of naïve retina

**Figure 7:**
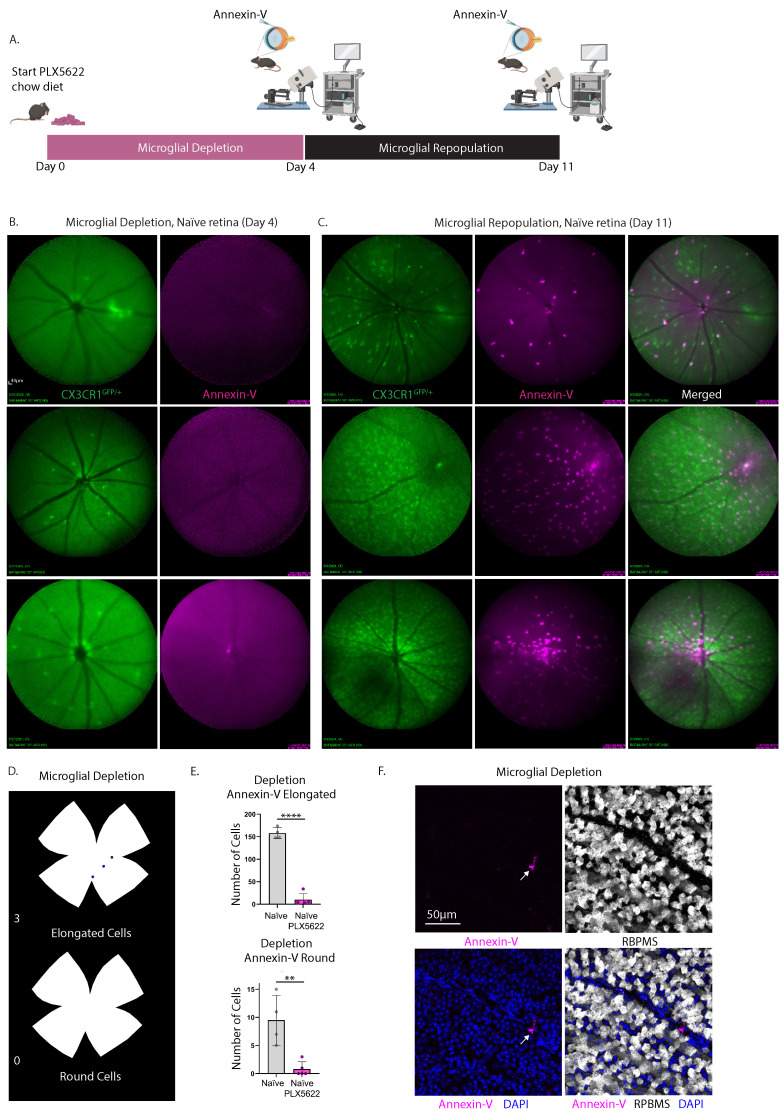
Experimental design describing the time course of microglial depletion and repopulation and cSLO fundus images taken at select time points to demonstrate that the bright annexin-V signals label a subset of microglia. **A.** Three naïve CX3CR1^GFP/+^ mice were provided a PLX5622-enriched chow diet (day 0) for a duration of 4 days. Following this treatment, mice underwent intravitreal injection with annexin-V and were subsequently imaged using cSLO under fluorescein angiography (FA) and Indocyanine Green angiography (ICGA) modes. The diet was then reverted back to standard chow to facilitate microglial repopulation for an additional 7 days. Retinal imaging was repeated as previously described. A total of 5 eyes were included in the study. One eye from an individual animal was excluded from imaging due to cataract formation resulting from lens trauma during the intravitreal injection. **B.** Simultaneous cSLO imaging was performed to visualize microglia (green) and annexin-V^+^ cells (magenta) at day 4, following microglial depletion. **C.** Simultaneous cSLO imaging of microglia (green) and annexin-V^+^ cells (magenta) was conducted after 7 days of microglial repopulation (day 11). Scale bar: 40µm. (Right) Overlay of simultaneous FA+ICGA acquisition mode of CX3CR1^GFP/+^ control mouse with annexin-V labeled cells. **D.** Isodensity maps illustrate the spatial distribution of annexin-V^+^ cells classified by morphology (elongated vs. round) after 4 days of microglial depletion with PLX5622. **E.** Quantification of annexin-V^+^ cells exhibiting either elongated or round morphology after 4 days of microglial depletion (n=5 eyes) was compared to naïve retina (no depletion, n=4 eyes). Data are expressed as mean ± standard deviation (SD). Statistical significance was determined using Student's t-test, with **p<0.01 and ****p<0.0001 indicating significant changes. **F.** A confocal image shows an individual annexin-V^+^ microglia (magenta), demonstrating that while PLX5622 significantly reduces their abundance, occasional annexin-V^+^ microglia remain. Ganglion cells are labeled with RBPMS (white) and nuclei are counterstained with DAPI (blue). Scale bar: 50µm.

To demonstrate that annexin-V visualized through cSLO fundus imaging labels a subset of microglia we assessed the impact of pharmacological microglia depletion on the annexin-V signals (**Fig. 7A**). CXCR1^GFP/+^ mice received a chow diet mixed with 1200mg/kg PLX-5622, a selective CSF1R inhibitor, for 4 days and acquired cSLO images of both GFP^+^ microglial cells and annexin-V. PLX5622 treatment depleted over 90% of the retinal microglia (**Fig. 7B, green**) and abolished annexin-V signals (**Fig. 7B, magenta**) in naïve retinas. Following depletion, normal chow was provided for 7 days to allow for microglial repopulation.

Upon repopulation, annexin-V+ cells (**Fig. 7C**, magenta) reappeared in proportion to the number of repopulated microglia (**Fig. 7C**, green), indicating a direct correlation between annexin-V positivity and microglial presence. Fundus images from the same eye before and after repopulation, using the vasculature for tracking (**Fig. 7B-C**), revealed that the returned annexin-V^+^ cells were CX3CR1^+^ microglia (**Fig. 7C right**). Notably, annexin-V signal was concentrated in the central retina near the ONH, suggesting possible contributions from extra-retinal sources like hyalocytes in the vitreous 30, 31 which can be a valuable indicator of ocular pathologies 32.

*Ex vivo* analysis of retinas (from a separate group of mice) after 4 days of microglial depletion showed a significant reduction in both elongated and round annexin-V^+^ cells (**Fig. 7D**). Quantitative analysis from n=5 eyes indicated a significant decrease in annexin-V+ cells (elongated, ****p<0.0001; round, **p<0.01) following microglial depletion compared to naïve retinas (n=4). Confocal imaging revealed occasional annexin-V^+^ cells, consistent with incomplete depletion due to residual microglia (**Fig. 7F**).

### Bright Annexin-V labeling following optic nerve crush is eliminated by pharmacological depletion of microglia revealing faint apoptotic labeling

**Figure 8:**
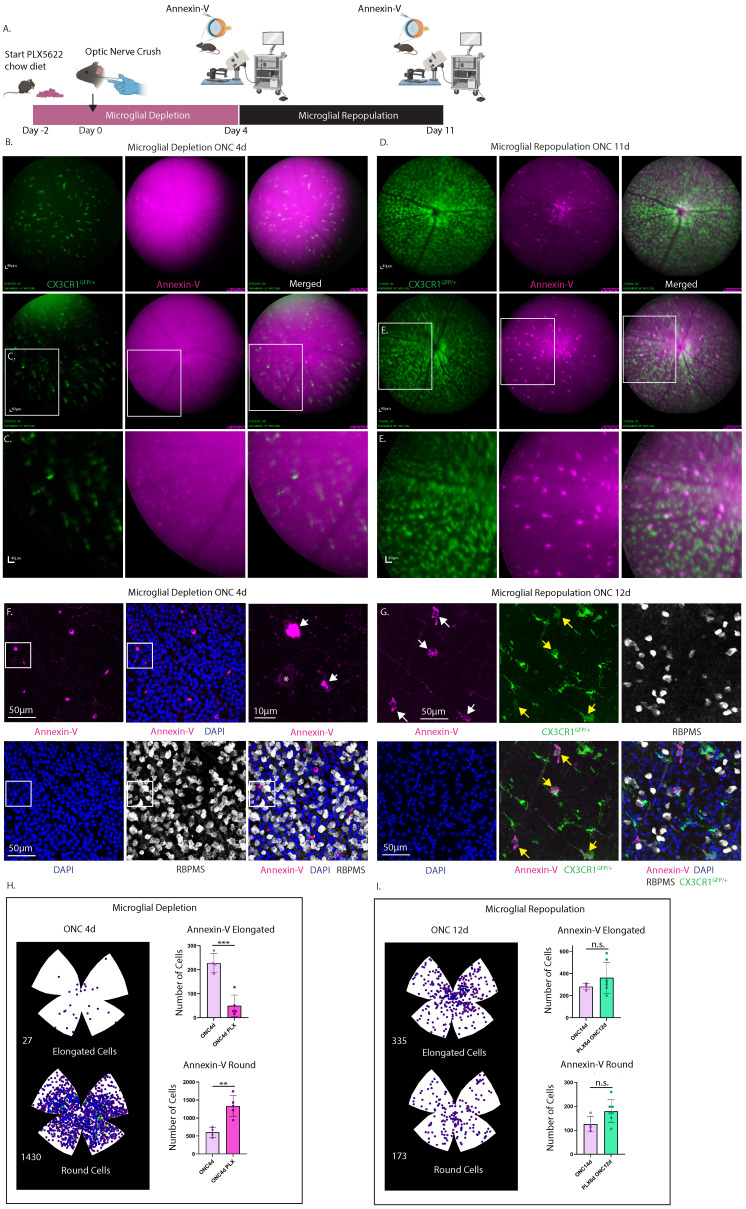
Experimental design describing time course of microglial depletion in optic nerve crush injury model followed by microglial repopulation. **A.** A group of CX3CR1^GFP/+^ mice (n=4) were administered PLX5622 chow starting 2 days prior to optic nerve crush injury (denoted as day 0). The PLX5622 diet was continued until 4 days post-ONC (ONC4d), shown in pink. **B.** At this time point (ONC4d, microglial depletion) cSLO was performed following intravitreal injection of annexin-V. Obtained images showed faint labeling of round cells, presumed to be apoptotic RGCs, which did not co-localize with CX3CR1. Mice were switched to a standard rodent chow diet for an additional 7 days (Day 11 post-ONC) to allow for microglial repopulation. Scale bar: 40μm. **C.** Zoomed in image of round, faintly labeled annexin-V^+^ cells. Scale bar: 40µm. **D.** At ONC11d (microglial repopulation) cSLO images were taken following intravitreal injection with annexin-V. Brightly labeled annexin-V^+^ elongated cells reappeared, while the faintly labeled round annexin-V^+^ cells were no longer prominently visible. **E.** A zoomed-in image revealed the bright annexin-V^+^ elongated cells, showed co-localization with CX3CR1 (green). **F.** Representative confocal image of WT mouse (ONC4d) injected with annexin-V (magenta) following microglial depletion and immunostained for RBPMS (white) and counterstained with DAPI (blue). Scale bar: 50 µm. **F.** (Upper Right) Zoomed in image of selected area (white box), white arrows indicating round annexin-V^+^ cells. Note: Brightly labeled round annexin-V^+^ cells did not appear to co-localize with RBPMS, suggesting that RBPMS downregulation might precede latter stages of apoptosis. A faintly labeled round annexin-V^+^ cell (*) co-localized with RBPMS indicating this may be an early stage of apoptosis (see **Fig. S6** for further details). Scale bar: 10 µm. **G.** Confocal images of annexin-V^+^ cells (magenta) from retinas taken from CX3CR1^GFP/+^ mice after microglial repopulation (ONC 12d). Most annexin-V^+^ cells (white arrows) were positive for CX3CR1 (green) but exhibited lower CX3CR1 expression (yellow arrows) compared to microglia expressing CX3CR1 only. RGCs were labeled with RBPMS (white) and nuclei counterstained with DAPI (blue). Scale bar: 50µm. **H.** (Left) Isodensity maps show the distribution of annexin-V^+^ cells (both elongated and round) in retinas after microglial depletion (ONC4d). **H.** (Right) Quantification of the number of annexin-V^+^ elongated cells (top) and annexin-V^+^ round cells (bottom) in retinas from n=5 eyes subjected to microglial depletion for 6 days (ONC4d) compared to non-depleted controls (n=4 eyes, ONC4d). Error bars indicate SD. **p<0.01 and ***p<0.001, statistically significant change (Student's t-test). **I.** (Left) Isodensity maps of annexin-V^+^ cells (elongated and round) in retinas after microglial depletion followed by repopulation (ONC12d). **I.** (Right) Quantification of the number of annexin-V^+^ elongated cells (top) and annexin-V^+^ round cells (bottom) from n=7 eyes subjected to microglial depletion (for 6 days) followed by repopulation (for 8 days) (ONC12d). Quantification from non-depleted controls (n=4 eyes, ONC14d) are shown for comparison. Statistical significance was assessed using Student's t-test (error bars indicate SD; n.s. = not statistically significant).

To discern whether the increase in annexin-V^+^ labeling post-injury is attributable to RGC apoptosis or a subset of retinal microglia that bind annexin-V, we conducted a microglia depletion/repopulation experiment in CXCR1^GFP/+^ and WT mice subjected to ONC injury (**Fig. 8A and Fig. S5**). Mice were provided PLX5622 chow diet two days prior to ONC to facilitate depletion of microglia. In CXCR1^GFP/+^ mice PLX5622 diet was continued till ONC4d which corresponded to the peak of round annexin-V labeling from histology (**Fig. 6B**). cSLO images of annexin-V signals were taken of mice at ONC4d (**Fig. 8B-C**, depletion) and at ONC11d (**Fig. 8D-E**, repopulation). Contrary to the naïve retina, microglial depletion eliminated the bright annexin-V^+^ elongated cells revealing faint round annexin-V^+^ cells, presumably apoptotic RGCs (**Fig. 8C**). Upon repopulation CX3CR1^+^ microglia return, and a subset are brightly co-labeled with annexin-V, indicating that annexin-V can bind to repopulated myeloid cells too. Similar results were obtained in WT mice subjected to longer PLX5622 depletion and imaged at ONC8d (depletion) and ONC15d (repopulation) (**Fig. S5**). These results were also corroborated by confocal microscopy of retinal flat mounts of WT mice intravitreally injected with annexin-V (ONC4d) showing labeling of round annexin-V^+^ cells (magenta) (**Fig. 8F, S6**). The depletion of microglial cells enabled us to evaluate the dynamics of annexin-V binding to dying RGCs. Using confocal imaging of retinal whole mounts, we searched for and found an example of a weakly annexin-V labeled RBPMS^+^ retinal ganglion cell (indicated by the *) suggesting an early stage of apoptosis (**Fig. 8F**). Early stage annexin-V binding to apoptotic cells is only to the phosphatidylserine that is flipped to the outer leaflet of the plasma membrane (thus the weaker staining). At latter stages of apoptosis when the cell's membrane breaks down, annexin-V can stain the cells interior phosphatidylserine as well resulting in a brighter signal. It is worth noting that Brn3a and RBPMS appear to be reduced in expression in apoptotic RGCs during the latter stages of apoptosis (**Fig. 6A, 8F, and S6**) when annexin-V labeling is more pronounced. This coincides with fragmentation and degradation of the nucleus (**Fig. S6**). Thus, we can conclude that the annexin-V signals present after microglial depletion are attributed to labeling of apoptotic cells.

Confocal images of retinas from CXCR1^GFP/+^ mice after microglial repopulation (ONC12d) (**Fig. 8G**) revealed that annexin-V^+^ cells have lower CX3CR1 expression compared to repopulated microglial that only express CX3CR1 suggesting differences in activity. Following microglial depletion at ONC4d there is a significant reduction in the number of elongated cells (p<0.001) and a notable increase in the number of round cells (p<0.01) (**Fig. 8H**). These observations are consistent with the hypothesis that PLX5622-induced microglial depletion led to the removal of microglia that previously contributed to the bright annexin-V signals detected using cSLO. Consequently, the remaining fainter annexin-V labeling is likely indicative of apoptotic RGCs.

Interestingly, the repopulation of microglia in these animals resulted in an increase in the number of elongated and round annexin-V^+^ cells, reaching levels comparable to those observed at ONC14d in mice that did not undergo microglial depletion (**Fig. 8I**).

These findings confirm that while DARC imaging, initially developed to identify stressed or apoptotic cells shows limitations in this area, annexin-V remains a useful indicator of ocular pathologies 32, reflecting both microglial activity and potential changes in retinal health.

## Discussion

Annexin-V is widely recognized as a universal biomarker for quantifying apoptosis. However, our results indicate annexin-V labeling in the retina is not exclusively specific to phosphatidyl-serine exposure on dying cells. Specifically, annexin-V^+^ cells are notably concentrated at the ONH after injury, as well as in intact retinas, suggesting that these cells are not exclusively RGCs. Similar observations have been reported in other injury models, such as ocular hypertension in rats 8, 33.

We identified various immune cells including CX3CR1^+^ cells (likely dendritic cells) and infiltrating vitreous cells (hyalocytes) that appear to bind annexin-V and contribute to the *in vivo* fluorescent signals observed using cLSO. In addition to the presence of annexin-V^+^ cells in uninjured retinas, an increase in annexin-V^+^ elongated cells (many resembling star-shaped hyalocytes 34) and weaker stained round cells, likely apoptotic RGCs, was noted following ON injury.

We show that the time course of the immune response following ON injury alters the distribution of annexin-V binding to immune cells present in the retina. This is likely attributed to transient infiltration of macrophages/monocytes or expansion of annexin-V positive cells which may include microglia and dendritic cells. Interestingly, a previous report characterized the response of dendritic cells after ONC 35, and their temporal course resembles annexin-V^+^ elongated cells. The behavior of these annexin-V^+^ cells to stimulate cell migration, infiltration, or proliferation under pathophysiological conditions can be an important indicator of a pathological event.

Our cSLO images revealed two distinct levels of annexin-V^+^ labeling intensity *in vivo*: strong and faint. Anatomical analysis confirmed that cells with round morphology and faint membrane staining corresponded to apoptotic RGCs, while cells with strong annexin-V labeling were identified as myeloid cells, resembling hyalocytes (**Fig. 5 and Fig. S3**). Moreover, the temporal course of annexin-V labeling *in vivo* closely aligned with the observed time course of annexin-V^+^ elongated myeloid cells determined *ex vivo*.

Our pharmacological depletion studies demonstrate that removal of CX3CR1^+^ microglia in both naïve retinas and those subjected to ONC injury results in elimination of bright annexin-V^+^ signals. This confirms that the bright annexin-V labeling observed in cSLO images is predominantly due to non-apoptotic binding of annexin-V to a subpopulation of resident myeloid cells, challenging the notion of annexin-V as an exclusive apoptosis biomarker. Notably, the weaker annexin-V^+^ cells, indicative of apoptosis, are only observed *in vivo* after microglial depletion and ONC injury, likely due to the imaging limitations of cSLO, which operates within specific intensity thresholds. Consequently, the detection of low-abundance annexin-V binding associated with apoptosis may be masked by the higher affinity binding to microglia. Also, the presence of microglial cells may phagocytose dying annexin-V^+^ RGCs weakening their contribution to the cSLO signal.

Although we cannot precisely identify the annexin-V^+^ myeloid cells, they display specific characteristics: 1) They present either round/ameboid-like appearance or are elongated/star-shaped with pseudopodia (**Fig. S3**). 2) Their CX3CR1 expression is lower, and their skeleton length and branches are fewer than the general population of CX3CR1^+^ cells that are annexin-V^-^. 3) These cells are found in the nerve fiber layer, vitreous (**Fig. 3B**), and the INL resembling microglial cells. 4) They are myeloid in origin, as evidenced by their elimination with PLX5622 treatment. 5) Depletion of these cells does not impact RGC survival after acute injury 36. 6) They increase in number in response to injury, though it remains unclear whether this increase is a consequence or cause of RGC loss. 7) Some annexin-V^+^ cells appear to engage in phagocytic clearance, as they are observed wrapping around and engulfing RGCs (**Fig. S2A**). 8) Their numbers peak around ONC8d and remain elevated through ONC21d, indicating a role in the later stages of injury response.

Further characterization of this annexin-V^+^ myeloid subpopulation is warranted to determine their role in injury responses and potential remodeling of the inflammatory microenvironment. It will also be valuable to explore whether this subtype is present in other tissues and if their presence could serve as an indicator of disease progression.

To date, the misnomer DARC (“Detection of Apoptosing Retinal Cells”) imaging has been described as a method to non-invasively identify apoptotic cells in the retina *in vivo*. Although our study is comparable to previous published reports of DARC imaging in rodent models, one limitation of our study is that annexin-V is solely delivered through intravitreal injection whereas intravenous delivery of annexin-V could result in different binding outcomes.

The discovery presented here, that annexin-V binds to immune cells in the retina and in the vitreous challenges the interpretation of DARC counts and the role of annexin-V in the diagnosis of retinal diseases. Although we only tested the naïve condition and in response to optic nerve crush injury, it is likely that DARC counts reflect microglial involvement in other retinal diseases as well. Thus, chronic retinal inflammation or retinal degenerative diseases pose an unresolved challenge in using annexin-V to evaluate apoptosis. Unlike the high rate of apoptosis following ONC, chronic pathologies are generally characterized by slower, progressive rates of cell death that may be difficult to observe by cSLO if they are sparse or outside the plane of focus. Also, annexin-V labeling of apoptotic cells only offers a single-snapshot in time of the actual apoptotic events and does not show the extent of damaged cells (those that have not yet entered apoptosis) or those that have already degenerated and have been phagocytosed and removed. Annexin-V labeling of a subpopulation of retinal immune cells that masks the weaker labeled apoptotic cells only further complicates its clinical interpretation.

However, it's worth noting that microglia are among the first cells to respond to damage and to changes in their microenvironment. Microglial activation and inflammatory responses are also major factors contributing to the development of retinal degenerative diseases such as glaucoma. Thus, detection of a subpopulation of annexin-V^+^ retinal immune cells in response to injury or disease may still be clinically relevant rather than apoptotic cell number, and provides an opportunity to monitor potential therapeutics 37-40 targeting microglial activation and neuroinflammation in the retina.

## Materials and Methods

### Mouse strains

In conducting research using animals, the investigator(s) adheres to the laws of the United States and regulations of the Department of Agriculture. All experiments and animal care were conducted according to protocols approved by the Animal Care and Use Committee of the National Eye Institute at the National Institutes of Health.

Wild type male and female C57BL/6J and CX3CR1^GFP/+^ mice (ages 3-6 months) used in this study were bred in our animal facility. Animal cages were selected at random and within each cage the animals were randomly assigned to experimental groups. All transgenic animals were confirmed by genotyping and the RD8 mutation was excluded by gene sequencing.

### Optic nerve crush

All surgical procedures were performed using aseptic procedures in the animal facility requiring masks, disposable lab coats, hair covers, sterile gloves, autoclaved instruments, and aseptic techniques. Animals were anesthetized with i.p. injection of ketamine (100mg/kg) / xylazine (6mg/kg) mixture. Eyes were protected from corneal dessication during the surgical procedure by application of Systane Ultra Lubricant Eye Drops (Alcon, Fort Worth, TX). Topical anesthesia (proparacaine hydrochloride ophthalmic solution) was provided to the eye undergoing ONC. A small incision was made in the conjunctiva at the lateral side of the left eyes (right eyes were used as control). Using forceps in one hand, the conjunctiva was pulled in the nasal direction to retract the eye. The white ON at the back of the eye was exposed and crushed with cross-action forceps for 10s approximately 0.5mm from the eye. Topical antibiotic (Neomycin, Polymyxin B sulfates, and Bacitracin Zinc Ophthalmic ointment, Bausch and Lomb Inc.) was provided to prevent infection. Mice were provided i.p. injection of Ketoprofen (5mg/kg) as part of the post-op analgesia. Animals were placed back in their home cages on top of heating pads and monitored during the recovery period until the animal was alert and ambulatory. Cages were flagged for veterinary observation for pain and distress per facility guidelines. Animals were euthanized with CO2 at various time points following ONC (2d, 4d, 8d, 14d, 21d).

### Microglial depletion and repopulation

We employed three experimental approaches for the depletion and repopulation of microglia in the retina. The first approach involved the use of naïve CX3CR1^GFP/+^ mice in which rodent chow (mixed with 1200mg/kg PLX-5622, Chemgood) was provided *ad libitum* for 4 days to deplete >90% of microglia 41. After 4 days of microglial depletion the animals were intravitreally injected with annexin-V to simultaneously image the status of both the microglia and the annexin-V^+^ cells. Animals were switched to normal rodent chow for 7 additional days to allow for microglial repopulation. The animals were then again intravitreally injected with annexin-V and reimaged on the cSLO. The second approach involved dietary administration of PLX5622 to CX3CR1^GFP/+^ mice beginning 2 days prior to optic nerve crush injury (injury date was designated day 0) and continuing until ONC4d. At ONC4d, animals were intravitreally injected with annexin-V to image the status of the annexin-V^+^ cells. Animals were then switched back to normal rodent chow for an additional 7 days to allow for microglial repopulation. At ONC11d, the animals were reinjected with annexin-V and reimaged on the cSLO. The third approach involved dietary administration of PLX5622 to WT mice beginning 2 days prior to optic nerve crush injury (injury date was designated day 0) and continuing until ONC8d. At ONC8d, animals were intravitreally injected with annexin-V to image the status of the annexin-V^+^ cells. Animals were then switched back to normal rodent chow for an additional 7 days to allow for microglial repopulation. At ONC15d, the animals were reinjected with annexin-V and reimaged on the cSLO.

### *In vivo* imaging

In preparation for *in vivo* imaging, animals were temporarily placed under anesthesia via inhalation of isoflurane (3-4% induction and 2-3% maintenance) with topical application of proparacaine to numb the eye prior to intravitreal injection of Annexin-V (1.25µL, 0.05 µg/µL or 0.2 µg/µL, CF@800, Biotium Inc., Cat#: 29078, CF@647, Biotium Inc., Cat#: 29003R-5ug. Any animals in which the injection caused lens opacity were excluded from imaging.

Animals were transported to the Heidelberg Spectralis HRA+OCT system (Heidelberg Engineering, Franklin, MA) for non-invasive, high resolution (1536 × 496) cSLO OCT imaging using the Indocyanine Green Angiography (ICGA) and Fluorescein Angiography (FA) settings. A 55-degree lens was used for the OCT scan and each b-scan image was averaged from 25 frames to improve signal-to-noise. cSLO fundus scans were averaged from 100 frames.

For *in vivo* imaging mice were anesthetized with inhalation of isoflurane delivered to effect in concentrations of 2-4% in oxygen (up to 5% for initial induction). Mydriasis was induced with 1% tropicamide (Akorn Inc., Lake Forest, Illinois) and 2.5% Phenylephrine Hydrochloride Ophthalmic Solution (Akorn Inc., Lake Forest, Illinois). GenTeal Gel (Alcon, Fort Worth, TX) was applied to each eye to prevent dessication.

### Tissue preparation and Immunofluorescence

Following euthanasia, eyes were enucleated and fixed in 4% paraformaldehyde in 1x PBS for 1h. The eyes were then washed in PBS and the retinas were dissected. Each retina was incubated with primary antibodies for either Brn3a^28^, ^29^ (C-20) (1:500, Santa Cruz Biotechnologies, Cat#: sc-31984), RBPMS^29^ (1:500, Genetex, Cat#: GTX118619), IBA1^42^ (1:500, EnCor Biotechnology, Cat#: CPCA-IBA1) or (1:500, Wako, Cat#: 019-19741), or MHC-II (1:500, Abcam, Cat#: ab139365) for 1 day at RT. For labeling of retina vasculature and activated microglia we incubated retinas with isolectin GS-IB4^43^ (1:200, Thermo Fisher Scientific, Cat#: I21412). For anterograde tracing of retinal projections, intravitreal injection of 1.2ul of cholera toxin subunit B (CTB)^44^ (1mg/ml, Thermo Fisher Scientific, Cat#: C34776) was used 3 days before tissue collection. The retinas were then washed in 1x PBS three times and stained overnight with secondary antibodies (Jackson ImmunoResearch Laboratories, 1:500). Each retina was then washed 3x with 1x PBS and then mounted onto glass slides with mounting medium ganglion side up.

### Quantification of retinal ganglion cells

Quantification of Brn3a^+^ RGCs was performed on entire retinas using a previously established algorithm written in ImageJ 45. Annexin-V^+^ cells were manually dotted by an experienced researcher that was blinded to the study using retinal photomontages in Photoshop 21.2.4 (Adobe Systems). In both instances, the ImageJ software automatically retrieved the cell/dot counts and their corresponding coordinates (x, y). Subsequently, this data was exported to a spreadsheet for further spatial analysis.

### Microglia morphology analysis

Images of microglia were converted to 8 bit grayscale images and analyzed in ImageJ 46. Briefly, brightness/contrast was adjusted to visualize the microglia processes and an unsharp mask filter was added to increase contrast. Despeckle was used to remove noise and the image was then converted to binary using threshold. Binary close was used to connect pixels separated by up to 2 pixels followed by the remove outliers function. Skeletonize was used to create the initial skeleton images which were than manually checked and corrected in Photoshop. Branch points (nodes) were dotted in Photoshop for quantification 45. Microglia process lengths were measured by quantifying the total number of white pixels in the image (single microglia analyzed at a time). The pixels were converted to µm using the scale bar. To quantify differences in CX3CR1 expression, a mask around the microglia cell body and processes was drawn and the resulting gray mean value was used for comparison. The difference in gray mean values were visually displayed by using the Rainbow RGB lookup table and by adjusting the brightness and contrast to utilize the full color spectrum.

### Spatial analysis

To evaluate spatial distribution and density, the number of neighbors per cell was computed using a fixed radius (70μm for Brn3a^+^ RGCs, both round and elongated Annexin-V^+^ cells), following a methodology previously outlined 45, and analyzed using SigmaPlot 13.0 for Windows (Systat Software). Cell colors indicate number of neighbors within a given radius. For Brn3a^+^ RGCs, the color scale ranged from 0-4 (purple) to 40-45 neighbors/RGC (dark red), with each color representing an increase of 5 neighbors. Meanwhile, the color scale for annexin-V^+^ cells ranged from 0 (purple) to 5 neighbors/annexin-V^+^ cell (yellow), with each color denoting an increase of one neighbor.

### Statistical analysis

Statistical comparisons of the number of surviving RGCs (Brn3a^+^), number of annexin^+^ (round or elongated shape) were performed in GraphPad Prism v8.3. Sample sizes were determined based on prior characterization of ONC in mice which results in a highly reproducible time course of RGC death. Groups were compared by one-way analysis of variance (multiple comparisons) with Tukey's post hoc test correction. Data are presented as mean ± standard deviation. For Brn3a, naïve (n=5 eyes), ONC2d (n=3 eyes), ONC4d (n=4 eyes), ONC8d (n=4 eyes), ONC14d (n=4 eyes), ONC21d (n=5 eyes). For Annexin-V round or elongated, naïve (n=4 eyes), ONC2d (n=3 eyes), ONC4d (n=4 eyes), ONC8d (n=3 eyes), ONC14d (n=4 eyes), ONC21d (n=5 eyes). Significance was considered at P<0.05. One animal was excluded from the ONC8d group annexin-V quantification because the annexin-V injection was not successful.

## Supplementary Material

Supplementary figures and tables.

## References

[1] Vermes I, Haanen C, Steffens-Nakken H, Reutelingsperger C (1995). A novel assay for apoptosis. Flow cytometric detection of phosphatidylserine expression on early apoptotic cells using fluorescein labelled Annexin V. J Immunol Methods.

[2] Koopman G, Reutelingsperger CP, Kuijten GA, Keehnen RM, Pals ST, van Oers MH (1994). Annexin V for flow cytometric detection of phosphatidylserine expression on B cells undergoing apoptosis. Blood.

[3] Fadok VA, Voelker DR, Campbell PA, Cohen JJ, Bratton DL, Henson PM (1992). Exposure of phosphatidylserine on the surface of apoptotic lymphocytes triggers specific recognition and removal by macrophages. J Immunol.

[4] Cordeiro MF, Guo L, Luong V, Harding G, Wang W, Jones HE (2004). Real-time imaging of single nerve cell apoptosis in retinal neurodegeneration. Proc Natl Acad Sci U S A.

[5] Guo L, Salt TE, Maass A, Luong V, Moss SE, Fitzke FW (2006). Assessment of neuroprotective effects of glutamate modulation on glaucoma-related retinal ganglion cell apoptosis *in vivo*. Invest Ophthalmol Vis Sci.

[6] Guo L, Salt TE, Luong V, Wood N, Cheung W, Maass A (2007). Targeting amyloid-beta in glaucoma treatment. Proc Natl Acad Sci U S A.

[7] Cordeiro MF, Normando EM, Cardoso MJ, Miodragovic S, Jeylani S, Davis BM (2017). Real-time imaging of single neuronal cell apoptosis in patients with glaucoma. Brain.

[8] Davis BM, Tian K, Pahlitzsch M, Brenton J, Ravindran N, Butt G (2017). Topical Coenzyme Q10 demonstrates mitochondrial-mediated neuroprotection in a rodent model of ocular hypertension. Mitochondrion.

[9] Davis BM, Pahlitzsch M, Guo L, Balendra S, Shah P, Ravindran N (2018). Topical Curcumin Nanocarriers are Neuroprotective in Eye Disease. Sci Rep.

[10] Guo L, Davis BM, Ravindran N, Galvao J, Kapoor N, Haamedi N (2020). Topical recombinant human Nerve growth factor (rh-NGF) is neuroprotective to retinal ganglion cells by targeting secondary degeneration. Sci Rep.

[11] Normando EM, Yap TE, Maddison J, Miodragovic S, Bonetti P, Almonte M (2020). A CNN-aided method to predict glaucoma progression using DARC (Detection of Apoptosing Retinal Cells). Expert Rev Mol Diagn.

[12] Cordeiro MF, Hill D, Patel R, Corazza P, Maddison J, Younis S (2022). Detecting retinal cell stress and apoptosis with DARC: Progression from lab to clinic. Prog Retin Eye Res.

[13] Corazza P, Maddison J, Bonetti P, Guo L, Luong V, Garfinkel A (2021). Predicting wet age-related macular degeneration (AMD) using DARC (detecting apoptosing retinal cells) AI (artificial intelligence) technology. Expert Rev Mol Diagn.

[14] Euzger HS, Flower RJ, Goulding NJ, Perretti M (1999). Differential modulation of annexin I binding sites on monocytes and neutrophils. Mediators Inflamm.

[15] Rosengarth A, Rösgen J, Hinz HJ, Gerke V (1999). A comparison of the energetics of annexin I and annexin V. J Mol Biol.

[16] Moss SE (1997). Annexins. Trends Cell Biol.

[17] Moss SE, Morgan RO (2004). The annexins. Genome Biol.

[18] Gerke V, Moss SE (2002). Annexins: from structure to function. Physiol Rev.

[19] Jung S, Aliberti J, Graemmel P, Sunshine MJ, Kreutzberg GW, Sher A (2000). Analysis of fractalkine receptor CX(3)CR1 function by targeted deletion and green fluorescent protein reporter gene insertion. Mol Cell Biol.

[20] Maass A, von Leithner PL, Luong V, Guo L, Salt TE, Fitzke FW (2007). Assessment of rat and mouse RGC apoptosis imaging *in vivo* with different scanning laser ophthalmoscopes. Curr Eye Res.

[21] Sánchez-Migallón MC, Valiente-Soriano FJ, Nadal-Nicolás FM, Vidal-Sanz M, Agudo-Barriuso M (2016). Apoptotic Retinal Ganglion Cell Death After Optic Nerve Transection or Crush in Mice: Delayed RGC Loss With BDNF or a Caspase 3 Inhibitor. Invest Ophthalmol Vis Sci.

[22] Murenu E, Gerhardt MJ, Biel M, Michalakis S (2022). More than meets the eye: The role of microglia in healthy and diseased retina. Front Immunol.

[23] Nadal-Nicolás FM, Jiménez-López M, Salinas-Navarro M, Sobrado-Calvo P, Vidal-Sanz M, Agudo-Barriuso M (2017). Microglial dynamics after axotomy-induced retinal ganglion cell death. J Neuroinflammation.

[24] Meghraoui-Kheddar A, Barthelemy S, Boissonnas A, Combadière C (2020). Revising CX3CR1 Expression on Murine Classical and Non-classical Monocytes. Front Immunol.

[25] Wolf J, Boneva S, Rosmus DD, Agostini H, Schlunck G, Wieghofer P (2022). Deciphering the Molecular Signature of Human Hyalocytes in Relation to Other Innate Immune Cell Populations. Invest Ophthalmol Vis Sci.

[26] Narumi M, Nishitsuka K, Yamakawa M, Yamashita H (2015). A survey of vitreous cell components performed using liquid-based cytology. Acta Ophthalmol.

[27] Greter M, Lelios I, Croxford AL (2015). Microglia Versus Myeloid Cell Nomenclature during Brain Inflammation. Front Immunol.

[28] Nadal-Nicolás FM, Jiménez-López M, Sobrado-Calvo P, Nieto-López L, Cánovas-Martínez I, Salinas-Navarro M (2009). Brn3a as a marker of retinal ganglion cells: qualitative and quantitative time course studies in naive and optic nerve-injured retinas. Invest Ophthalmol Vis Sci.

[29] Nadal-Nicolás FM, Galindo-Romero C, Lucas-Ruiz F, Marsh-Amstrong N, Li W, Vidal-Sanz M (2023). Pan-retinal ganglion cell markers in mice, rats, and rhesus macaques. Zool Res.

[30] Huang Y, Xu Z, Xiong S, Qin G, Sun F, Yang J (2018). Dual extra-retinal origins of microglia in the model of retinal microglia repopulation. Cell Discov.

[31] Wieghofer P, Engelbert M, Chui TY, Rosen RB, Sakamoto T, Sebag J (2022). Hyalocyte origin, structure, and imaging. Expert Rev Ophthalmol.

[32] Vagaja NN, Chinnery HR, Binz N, Kezic JM, Rakoczy EP, McMenamin PG (2012). Changes in murine hyalocytes are valuable early indicators of ocular disease. Invest Ophthalmol Vis Sci.

[33] Yap TE, Davis BM, Guo L, Normando EM, Cordeiro MF (2018). Annexins in Glaucoma. Int J Mol Sci.

[34] Migacz JV, Otero-Marquez O, Zhou R, Rickford K, Murillo B, Zhou DB (2022). Imaging of vitreous cortex hyalocyte dynamics using non-confocal quadrant-detection adaptive optics scanning light ophthalmoscopy in human subjects. Biomed Opt Express.

[35] Lehmann U, Heuss ND, McPherson SW, Roehrich H, Gregerson DS (2010). Dendritic cells are early responders to retinal injury. Neurobiol Dis.

[36] Hilla AM, Diekmann H, Fischer D (2017). Microglia Are Irrelevant for Neuronal Degeneration and Axon Regeneration after Acute Injury. J Neurosci.

[37] Gui S, Tang W, Huang Z, Wang X, Gui S, Gao X (2023). Ultrasmall Coordination Polymer Nanodots Fe-Quer Nanozymes for Preventing and Delaying the Development and Progression of Diabetic Retinopathy. Adv Funct Mater.

[38] Cao F, Liang K, Tang WW, Ni QY, Ji ZY, Zha CK (2024). Polyvinylpyrrolidone-curcumin nanoparticles with immune regulatory and metabolism regulatory effects for the treatment of experimental autoimmune uveitis. J Control Release.

[39] Jiang Z, Liang K, Gao X, Cao F, An G, Gui S (2023). Fe-curcumin nanozyme-mediated immunosuppression and anti-inflammation in experimental autoimmune uveitis. Biomater Res.

[40] Cao F, Gui S-Y, Gao X, Zhang W, Fu Z-Y, Tao L-M (2022). Research progress of natural product-based nanomaterials for the treatment of inflammation-related diseases. Materials & Design.

[41] Dagher NN, Najafi AR, Kayala KM, Elmore MR, White TE, Medeiros R (2015). Colony-stimulating factor 1 receptor inhibition prevents microglial plaque association and improves cognition in 3xTg-AD mice. J Neuroinflammation.

[42] Ma W, Silverman SM, Zhao L, Villasmil R, Campos MM, Amaral J (2019). Absence of TGFβ signaling in retinal microglia induces retinal degeneration and exacerbates choroidal neovascularization. Elife.

[43] Ferraro G, Gigante Y, Pitea M, Mautone L, Ruocco G, Di Angelantonio S (2023). A model eye for fluorescent characterization of retinal cultures and tissues. Sci Rep.

[44] Yao F, Zhang E, Gao Z, Ji H, Marmouri M, Xia X (2018). Did you choose appropriate tracer for retrograde tracing of retinal ganglion cells? The differences between cholera toxin subunit B and Fluorogold. PLoS One.

[45] Xiao X, Zhao T, Miyagishima KJ, Chen S, Li W, Nadal-Nicolás FM (2021). Establishing the ground squirrel as a superb model for retinal ganglion cell disorders and optic neuropathies. Lab Invest.

[46] Young K, Morrison H (2018). Quantifying Microglia Morphology from Photomicrographs of Immunohistochemistry Prepared Tissue Using ImageJ. J Vis Exp.

