# Supplementary material for: Annexin-V binds subpopulation of immune cells altering its interpretation as an in vivo biomarker for apoptosis in the retina
Source: Int J Biol Sci. 2024 Nov 11;20(15):6073–89. doi: 10.7150/ijbs.102551 (PMC11628321; doi:10.7150/ijbs.102551)
Supplement: Supplementary file 1 — Supplementary figures and tables. [file ijbsv20p6073s1.pdf]

Figure S1

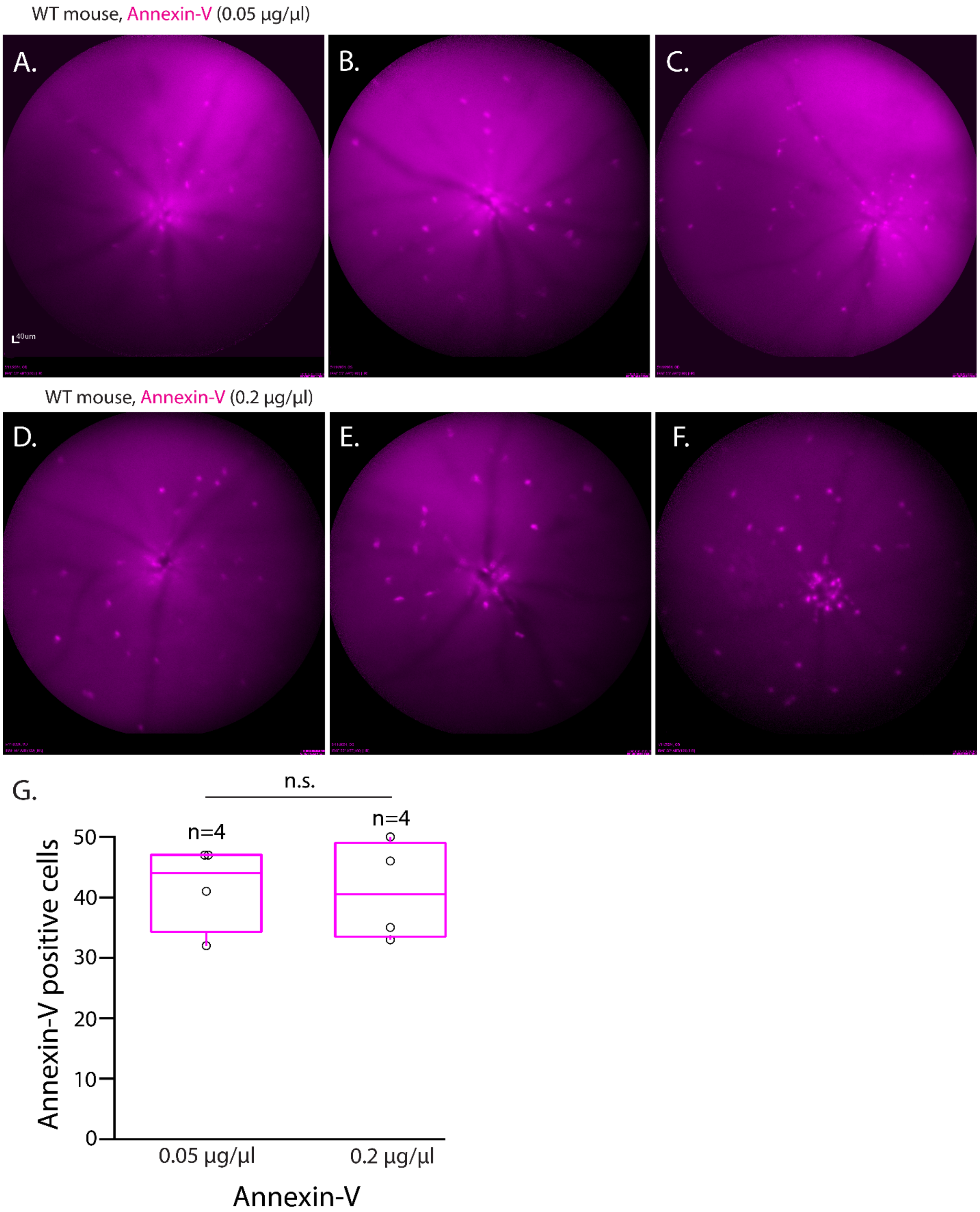

Figure S1. cSLO fundus images of naïve WT mice intravitreally injected with annexin-V to assess optimal concentration to use to uniformly label retinal cells. **A-C.** cSLO images of WT mice following intravitreal injection of annexin-V (0.05µg/µl). **D-F.** cSLO images of WT mice following intravitreal injection of annexin-V (0.2µg/ µl). Scale bar: 40µm. **G.** Quantification of annexin-V labeled cells shown as box plots. Each box represents the 25th (box bottom) to 75th (box top) percentiles. Lines inside the boxes represent the median values. The whisker top represents the 90th percentile and the whisker bottom represents the 10th percentile. No significant differences were observed between the lower dose annexin-V (0.05µg/µl) and the higher dose annexin-V (0.2µg/µl). We selected the higher concentration in all subsequent experiments to ensure reproducibility by offsetting for any injection site leakage.

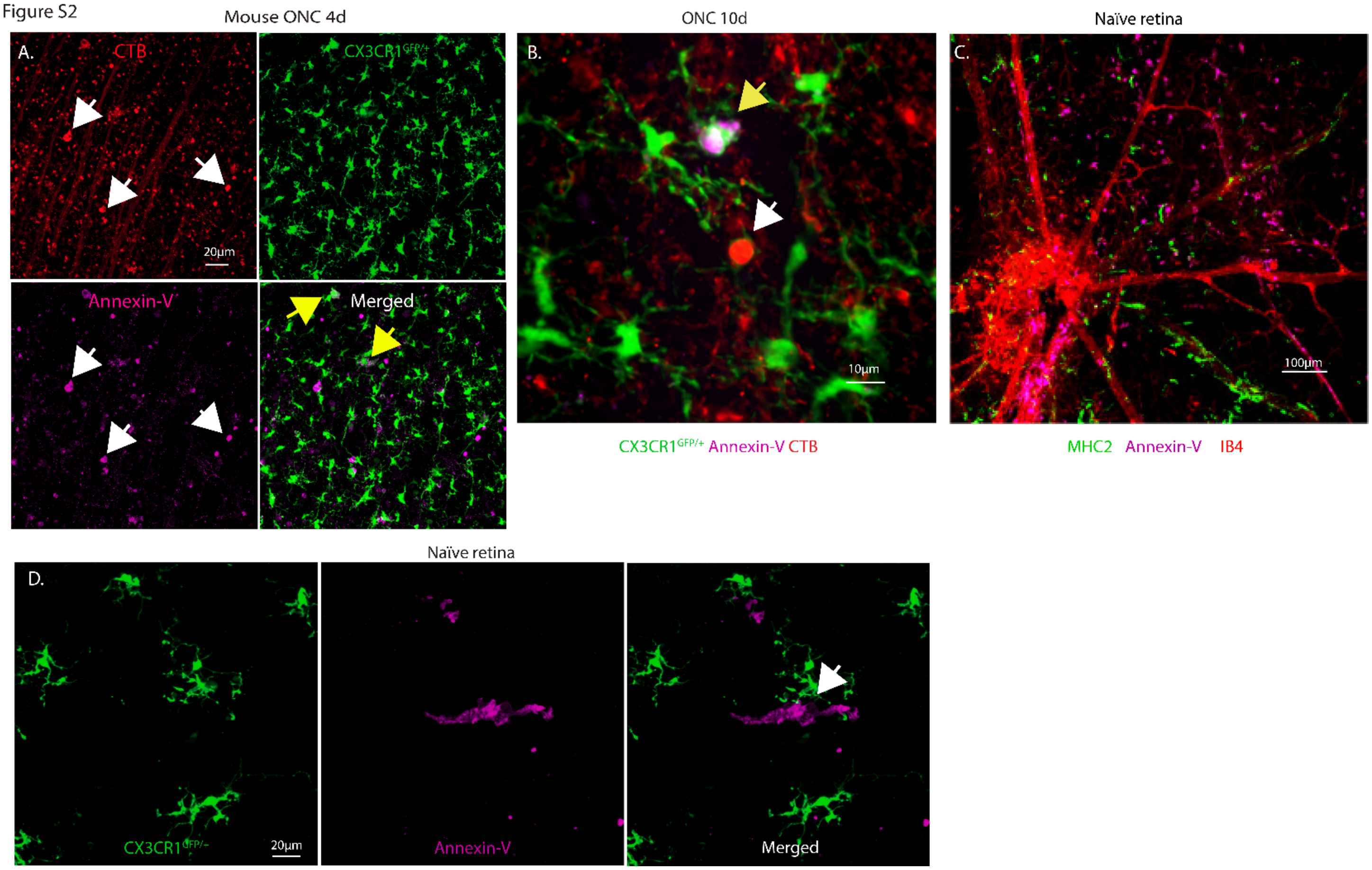

Figure S2. Representative confocal immunofluorescence images of retinal whole-mounts showing annexin-V labeling in relation to other retinal markers for ganglion cells, microglia, and retinal vasculature. **A.** In mice subjected to ONC, at 4d post-injury, co-localization of CTB and annexin-V was observed indicating the presence of apoptotic retinal ganglion cells (white arrows). Additionally, some CX3CR1^{+} microglia (green) exhibited annexin-V labeling (yellow arrows) that appear to engulf dying RGCs. Scale bar: 20µm. **B.** CX3CR1$^{GFP/+}$ mice were subjected to optic nerve crush injury with CTB injected intravitreally 3 days prior to retinal collection and annexin-V 1 hour. before collection. Retinas were harvested at 10days post-ONC. The image depicts a microglial cell labeled with annexin-V (yellow arrow) in proximity to a CTB labeled RGC (white arrow). Scale bar: 10µm. **C.** Naïve WT mice were intravitreally injected with annexin-V 1 hour prior to retinal dissection. The dissected retinas were immunostained for MHC class II (green) a marker associated with antigen presentation and microglial activation[40] and co-stained with IB4 (red) to label retinal vasculature and activated microglia. Annexin-V labeled microglia were observed on or near the vasculature, particularly close to the ONH. Scale bar: 100µm. **D.** In naïve retinas, a small proportion of annexin-V^{+} cells were observed to be CX3CR1^{-} (white arrow). Scale bar: 20µm.

Figure S3

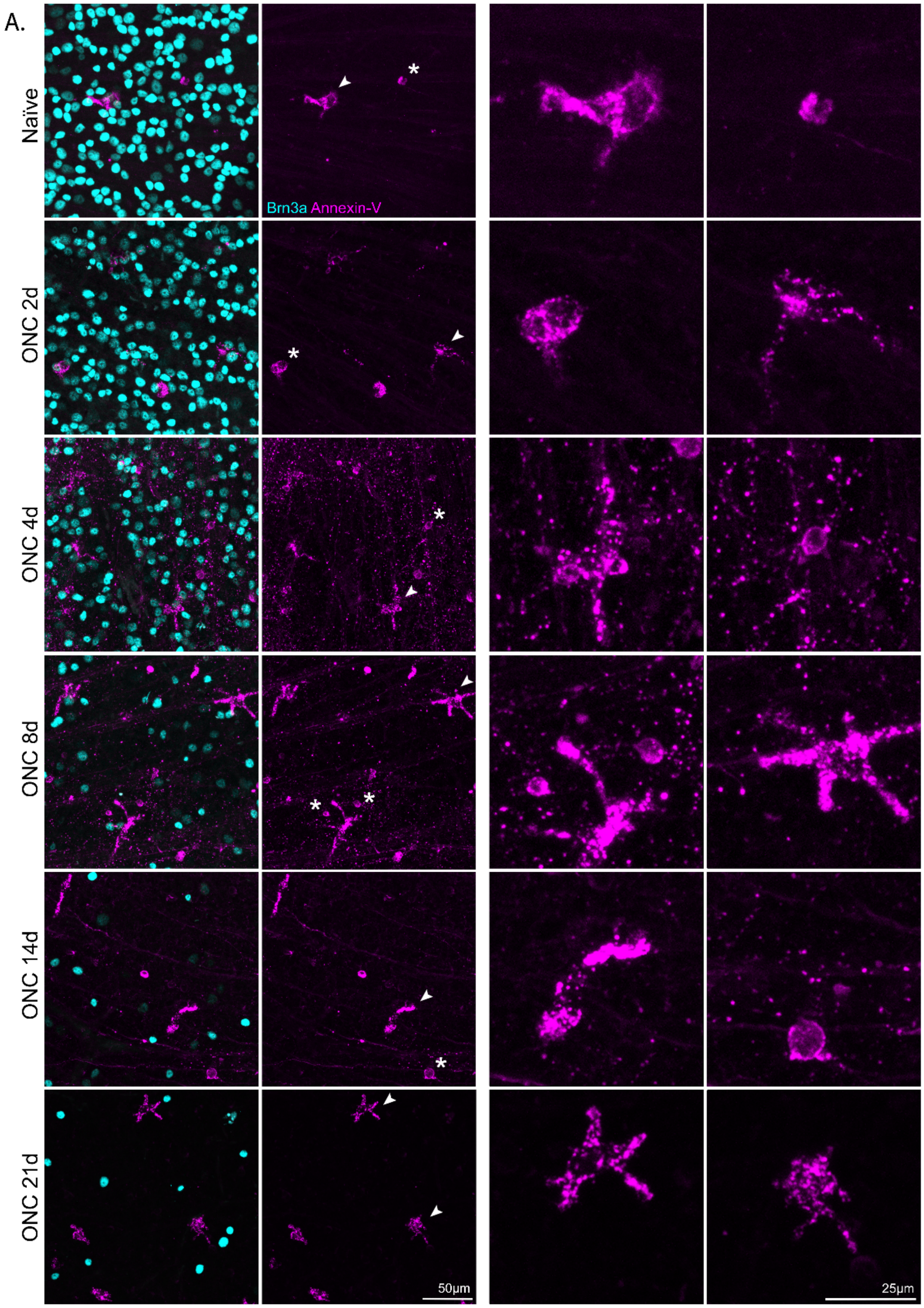

Figure S3. Representative confocal immunofluorescence images of retina before and after optic nerve crush injury at different time points showing morphological differences in annexin-V labeled cells (magenta) in relation to retinal ganglion cells (Brn3a, cyan). **A**. Naïve retina shows an example of an elongated annexin-V$^+$ cell (arrow) and a round annexin-V$^+$ cell (asterisk). Note the round cell has dense annexin-V labeling but lacks Brn3a expression. ONC2d shows an example of annexin-V$^+$ round cells phagocytosing Brn3a positive retinal ganglion cells (asterisk) and an elongated annexin-V$^+$ cell with short processes to the right. ONC4d shows an example of an elongated cell (arrow) with a distinctive star-shaped pattern with pseudopodia resembling hyalocytes [41]. To the upper right is a round membrane-bound annexin-V cell with a 'ghost-like' appearance (asterisk). These faintly labeled cells are likely apoptotic retinal ganglion cells. ONC8d shows round annexin-V$^+$ cells demarcated with an asterisk and a star-shaped cell (arrow) in the upper right. ONC14d shows an elongated annexin-V positive cell (arrow) and a round membrane bound cell undergoing apoptosis. At 21d several star-shaped annexin-V$^+$ cells are visible (arrows).

Figure S4

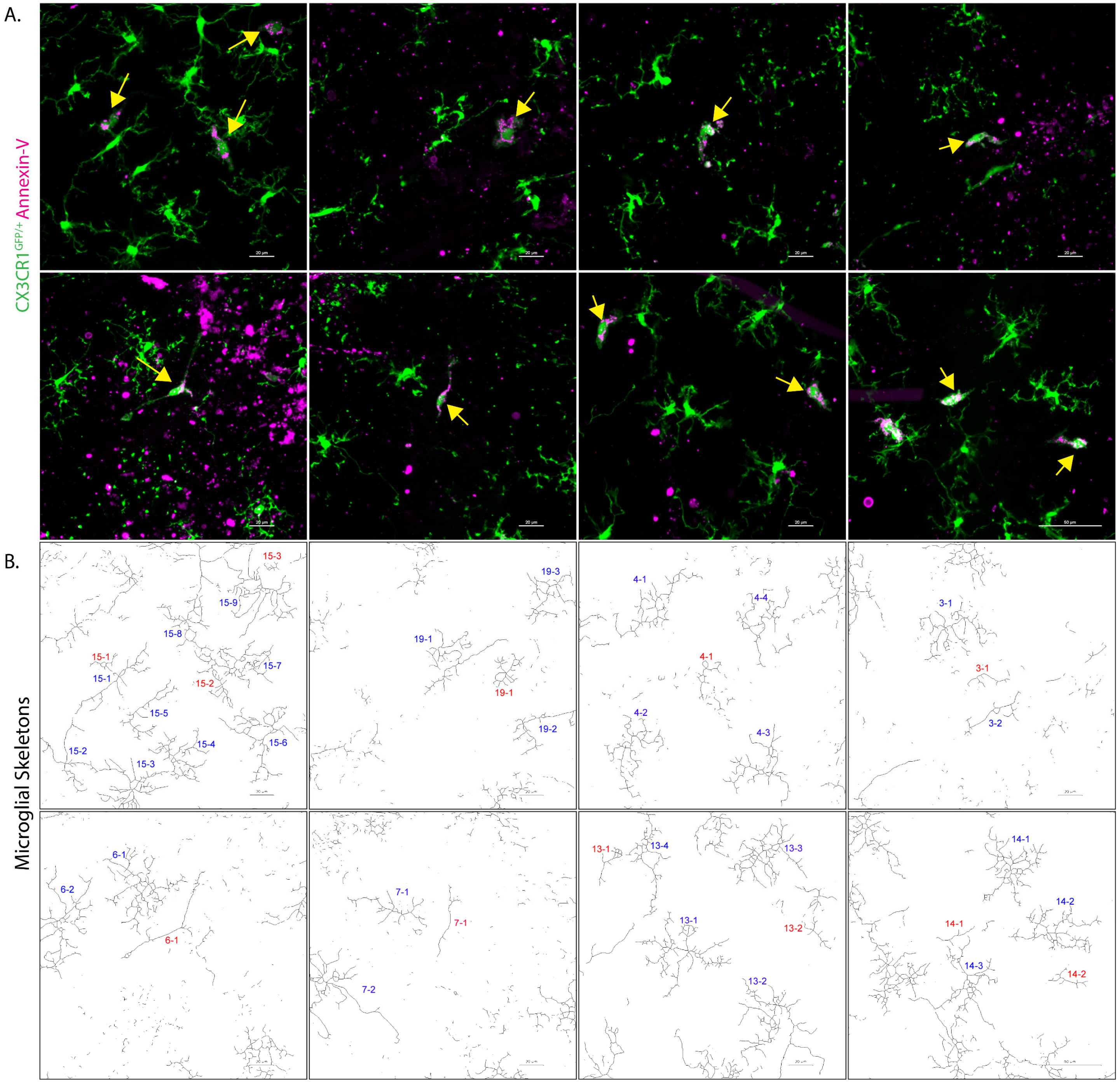

Figure S4. Confocal immunofluorescence images and matching skeletal analysis of retinal microglial morphology in naïve retinas. **A.** Maximum intensity projection images (n=8) of CX3CR1^{+} microglia (n=29) and CX3CR1^{+} Annexin-V^{+} (n=12) microglia (20x Magnification, 3x digital zoom). Scale bar: 20µm. **B.** Digital skeletons of microglial morphology taken from images shown in **A** and used for quantitative analysis (**Fig. 5E-F**).

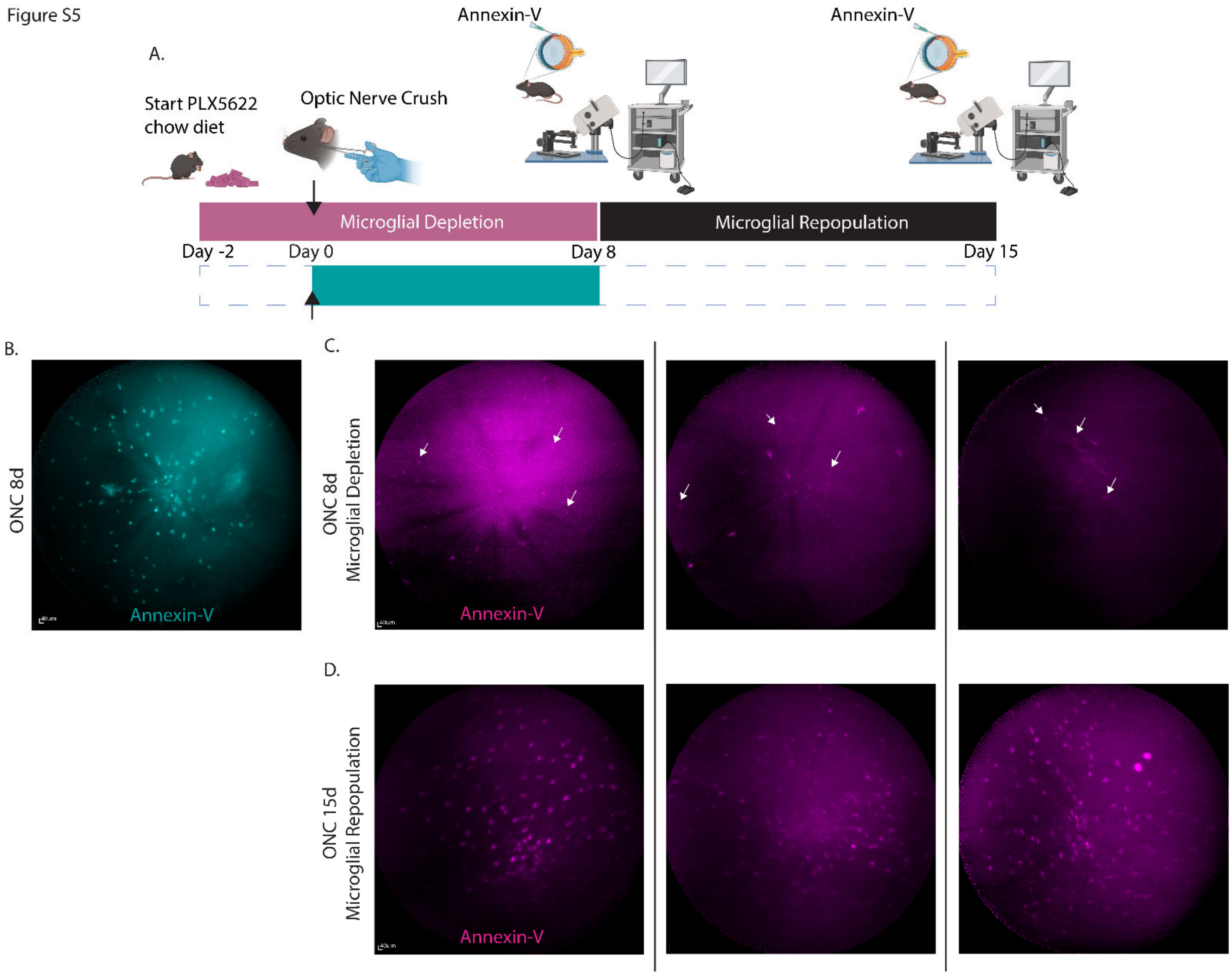

Figure S5. Experimental design outlining the time course of microglial depletion and subsequent repopulation in a murine model of optic nerve crush injury. **A.** Wild-type (WT) mice were divided into two groups. The first group (n=3) was administered a standard rodent chow and underwent optic nerve crush (ONC) on day 0, indicated by the cyan line. The second group (n=3) received PLX5622-containing chow beginning 2 days prior to ONC, continuing until 8 days post-ONC (ONC8d), as indicated by the pink line. **B.** Representative cSLO image of annexin-V (cyan) labeled cells shown for a WT mouse (group 1) at ONC8d. **C.** For mice in group 2 (microglial depletion), cSLO images were taken at ONC8d following intravitreal injection of annexin-V revealing a significant reduction in annexin-V labeling compared to cSLO image (no depletion) shown in **B**. Faintly labeled annexin-V^{+} cells can be seen (white arrows). **D.** Following the depletion phase, animals were transitioned to standard rodent chow for an additional 7 days (day 15 post-ONC) to allow for microglial repopulation. cSLO images taken after intravitreal annexin-V injection at ONC15d, show the return of annexin-V^{+} cells coinciding with microglial repopulation. Scale bar: 40μm.

Figure S6

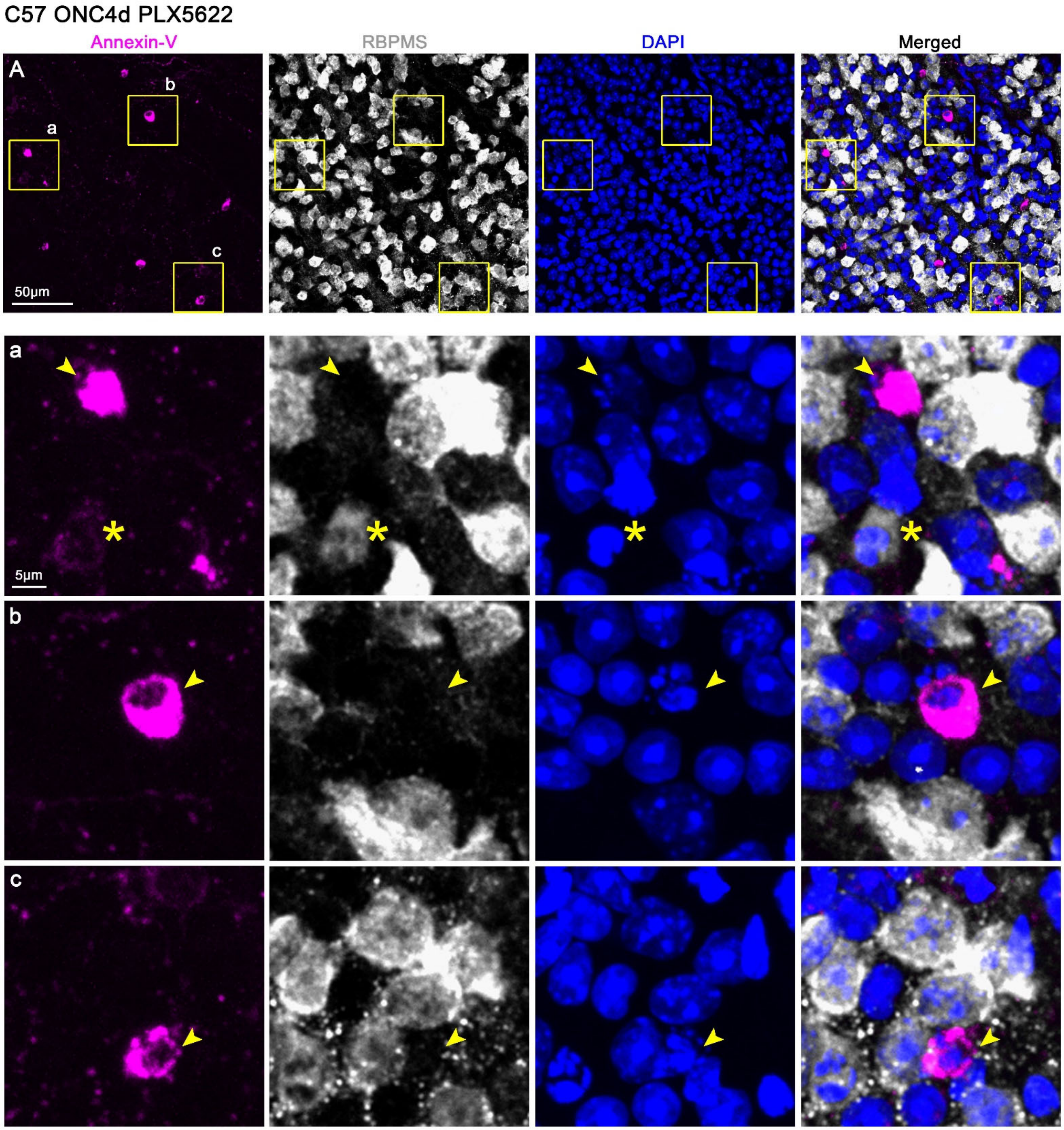

Figure S6. A. Annexin-V+ cells (magenta) selected from the original image (shown in Fig. 8F, ONC4d, microglial depletion). RGCs labeled with RBPMS (white) and nuclei counterstained with DAPI (blue). Scale bar: 50µm. Boxes are magnified in a, b, and c. **a.** In this region, two cells are highlighted with either an arrow or an asterisk. Arrow pointing at brightly labeled round annexin-V+ cell (apoptotic RGC, late-stage apoptosis) lacking RBPMS expression. DAPI staining reveals degraded nuclei. Asterisk indicates faintly membrane-labeled annexin-V+ cell (apoptotic RGC, early-stage apoptosis) that expresses RBPMS but shows sign of nuclear fragmentation. **b-c.** Arrows pointing at brightly labeled annexin-V+ cell (apoptotic RGC, late-stage apoptosis) with no RBPMS expression, as well as nuclei that appear disintegrated. Scale bar for zoomed images: 5µm.

Supplementary Table 1. Summary of in vivo annexin-V labeling before and after ONC (Mean ± SD).

	Control	2d	4d	8d	14d	21d
**Annexin-V$^+$ Cells**	46.8 ± 15.5	38.5 ± 8.7	124.6 ± 50.8	136.5 ± 71.8	127 ± 24.9	129.8 ± 26.4

Supplementary Table 2. Summary of ex vivo RGC and annexin-V labeling before and after ONC (Mean ± SD).

	Control	2d	4d	8d	14d	21d
**Brn3a$^+$**	44,863.2 ± 876.8	40,462 ± 1212.2	27,781 ± 2636	10,201 ± 603.9	4,051 ± 507.7	2,506.4 ± 329
**Annexin-V$^+$ Round**	9.5 ± 4.4	12.7 ± 4.9	602.8 ± 137.3	195.3 ± 46.07	126 ± 32.7	87.4 ± 26.8
**Annexin-V$^+$ Elongated**	157.8 ± 12.1	192.3 ± 43	227.8 ± 39.9	403.3 ± 92.0	281.8 ± 28.6	363.4 ± 77.3
